# *DeepPhysioRecon*: Tracing peripheral physiology in low frequency fMRI dynamics

**DOI:** 10.1162/IMAG.a.163

**Published:** 2025-09-25

**Authors:** Roza G. Bayrak, Colin B. Hansen, Jorge A. Salas, Nafis Ahmed, Ilwoo Lyu, Mara Mather, Yuankai Huo, Catie Chang

**Affiliations:** Department of Electrical and Computer Engineering, Vanderbilt University, Nashville, TN, United States; Department of Computer Science, Vanderbilt University, Nashville, TN, United States; Covera Health, New York, NY, United States; Department of Teaching and Learning, Peabody College, Nashville, TN, United States; Department of Computer Science, University of Waterloo, Waterloo, ON, Canada; Department of Computer Science and Engineering, Pohang University of Science and Technology, Pohang-si, Gyeongsangbuk-do, South Korea; Leonard Davis School of Gerontology, University of Southern California, Los Angeles, CA, United States; Department of Psychology, University of Southern California, Los Angeles, CA, United States; Department of Biomedical Engineering, University of Southern California, Los Angeles, CA, United States; Deparment of Biomedical Engineering, Vanderbilt University, Nashville, TN, United States

**Keywords:** brain-body, fMRI, respiration, heart rate

## Abstract

Many studies of the human brain using functional magnetic resonance imaging (fMRI) lack physiological measurements, which substantially impacts the interpretation and richness of fMRI studies. Natural fluctuations in autonomic physiology, such as breathing and heart rate, provide windows into critical functions, including cognition, emotion, and health, and can heavily influence fMRI signals. Here, we developed *DeepPhysioRecon*, a Long-Short-Term-Memory (LSTM)-based network that decodes continuous variations in respiration amplitude and heart rate directly from whole-brain fMRI dynamics. Through systematic evaluations, we investigate the generalizability of this approach across datasets and experimental conditions. We also demonstrate the importance of including these measures in fMRI analyses. This work highlights the importance of studying brain-body interactions, proposes a tool that may enhance the efficacy of fMRI as a biomarker, and provides widely applicable open-source software.

## 1 Introduction

The brain and body are closely coupled and continuously influencing each other. Brain-body interactions underpin key functions, including cognition and emotion, as well as the overall health of an organism (Azzalini et al., 2019; Barrett & Simmons, 2015; Koban et al., 2021; Shokri-Kojori et al., 2018).

Functional magnetic resonance imaging (fMRI) is a powerful and widely used technique in human brain research. While fMRI studies do not routinely incorporate physiological signals measured from the body, there is a growing trend toward acquiring continuous physiological measurements (such as heart rate and respiration) during fMRI scans. One motivation stems from considerations about interpretation and reproducibility of fMRI, a pressing issue in the neuroscience field (Botvinik-Nezer et al., 2020). Since fMRI is based on measurements of blood oxygenation, fMRI signals are influenced not only by spatially local changes in neural activity (Bandettini et al., 1992; Kwong et al., 1992; Ogawa et al., 1992), but also by any bodily physiological process that modulates blood oxygenation (Murphy et al., 2013). If not modeled, the presence of these additional effects can complicate the inferences drawn from fMRI as well as drive variability across results, such as in the mapping of large-scale brain networks (Birn et al., 2006; Xifra-Porxas et al., 2021).

Moreover, although physiological effects in fMRI are often regarded merely as confounds, several lines of work indicate that they also provide valuable information. For example, breathing and heart rate exert spatially structured, dynamic influences on cerebral blood oxygenation that closely mirror the spatial structure of core neuronal networks, suggesting a close connection (and potential interactions) between the regulation of blood flow and neuronal responses (Bright et al., 2020; Chen et al., 2020; Pinto et al., 2017). In fact, blood flow responses to heart rate variability have the potential to strengthen neural connectivity within networks involved in emotion regulation, highlighting the bidirectional connection between the brain and the body (Nashiro et al., 2023). Further, removing physiological components of fMRI signals has been shown to reduce test-retest reliability of individual differences in functional connectivity (Dubois & Adolphs, 2016).

Two major physiological drivers of the fMRI signal arise from natural, slowly varying (<0.15 Hz) fluctuations in respiration volume (RV) and heart rate (HR) (Birn et al., 2006; Shmueli et al., 2007). These fluctuations influence fMRI signals through multiple mechanisms, including changes in blood pressure (Katura et al., 2006), autonomic tone (Özbay et al., 2019; Picchioni et al., 2022), and arterial carbon dioxide (Birn et al., 2006; Wise et al., 2004). RV and HR variations are found to account for substantial variance in fMRI signals and to have a larger impact on brain functional connectivity measures compared to physiological effects that are directly synchronized with the breathing and cardiac cycles (Xifra-Porxas et al., 2021). Furthermore, the influence of RV and HR substantially overlaps with neurally mediated BOLD responses, spanning the same low frequency range (0.01–0.15 Hz) and overlapping with widely distributed functional networks (Birn et al., 2006). Therefore, the ability to precisely identify these low-frequency physiological components of BOLD fMRI data is crucial for their use as either ‘noise’ or valuable ‘signal’.

However, it is not always possible to acquire clean external physiological measures during fMRI (Glasser et al., 2018), and many existing datasets lack such measures altogether (ADNI (Jack et al., 2015); UK Biobank (Bycroft et al., 2018); HCP 7T Release (WU-Minn, 2017)). Accordingly, computational approaches have been developed for detecting RV and HR effects in the absence of physiological monitoring. Recent studies in young adults (Addeh et al., 2024; Bayrak et al., 2020; Salas et al., 2021) and in a pediatric cohort (Addeh et al., 2023) have demonstrated that RV signals can be reconstructed from fMRI data, but have either used window-based approaches that consider only short segments of the data at a time, or have not fully leveraged the potential of neural network architectures capable of processing complete sequences. Moreover, these studies did not also examine HR. Since HR has been associated with functional circuits for emotion regulation and correlates with dynamic variation in large-scale functional networks, the ability to reconstruct an HR signal directly from fMRI would contribute an additional rich source of information to fMRI studies. Methods have been proposed to infer cardiac phase information directly from fMRI (Ash et al., 2013; Aslan et al., 2019; Beall & Lowe, 2007; Colenbier et al., 2022), from which low-frequency variation in heart rate can be derived. These studies required either multiband sequences or examination of slice-specific signals, the latter of which requires data in native space where slice-wise timing is preserved. This work differs from these approaches by integrating RV and HR prediction into a single framework and by reconstructing HR from volume-wise data with conventional TR (>1 s). This unification offers two key benefits: It provides a more streamlined end-to-end solution and leverages the known covariation between RV and HR to improve the estimation of both physiological signals.

We have developed and rigorously evaluated a computational framework for inferring slow changes in respiratory volume and heart rate directly from the fMRI signal, without requiring fast multiband sampling or slice-based reconstructions. Motivated by the known coupling between respiratory and cardiac systems, we hypothesize that learning RV and HR simultaneously could boost the prediction accuracy. Therefore, we developed a framework that leverages multi-task learning (MTL). The present study builds on our initial work (Bayrak et al., 2021) by expanding the model validation with new experiments, larger subject pools, an external dataset, and a task model for brain-state dependency. Furthermore, we assess the utility of the predicted signals along with the interpretation and sensitivity of the model.

## 2 Methods

### 2.1 Datasets

#### 2.1.1 Overview

Models were trained using data from the Human Connectome Project Young Adult (HCP-YA) cohort (Van Essen et al., 2013). Written informed consent was obtained from participants, and the original study was approved by the Institutional Review Board of Washington University. Two categories of models were constructed: resting-state models (rs-models) and task models (t-models) (see Supplementary Table S1 for the list of training and testing dataset). Resting-state models were trained using HCP-YA resting-state data using five-fold cross-validation (CV), ensuring that subjects considered in the training phase were excluded from the testing. In addition, to evaluate the generalizability of rs-models across subjects both within (resting state) and between (task vs. resting state) conditions, rs-models were tested on both HCP-YA task data as well as a separate, in-house dataset with different acquisition parameters. Similarly, the t-models were trained on HCP-YA task datasets, and tested on held-out HCP-YA task data (with different task conditions), HCP-YA resting state data, and the in-house dataset. The individual datasets and acquisition parameters are described below.

#### 2.1.2 Dataset 1

A set of resting-state fMRI scans was drawn from the publicly available HCP 1200 subject release. fMRI scans in this release were acquired using a simultaneous multi-slice EPI sequence with the following parameters: TR = 0.72 s, duration of 14.33 mins, voxel size of 2 mm isotropic, TE = 33.1 ms, multi-band factor = 8, flip angle = 52 deg, and 72 slices. During the resting-state scans, subjects were instructed to keep their eyes open and fixate on a cross-hair, and subjects underwent four scans (two runs on 1 day, and two runs on a 2nd day). A subset of the HCP-YA resting-state dataset, consisting only of those subjects whose physiological signals were reported to have passed a quality assessment in both Power et al. (2020) and Xifra-Porxas et al. (2021), was included in this study. This procedure resulted in N = 375 subjects (with all four runs, totaling 1500 scans).

#### 2.1.3 Dataset 2

Our study also drew upon task fMRI scans from the HCP 1200 subject release. These scans were acquired with the same parameters as the resting-state fMRI data, described above, except for the duration of the scans. The task dataset comprises seven tasks: Working Memory, Social Cognition, Emotion Processing, Gambling, Relational Processing, Motor, Language. These tasks were designed to activate a variety of brain networks, and each subject underwent two scans per task (both runs on the same day).

All scans from the t-fMRI datasets were initially considered for model training and validation; however, only a subset of scans was ultimately used. A quality assessment was employed, using automated selection criteria to remove scans with poor-quality physiological data (this script and the list of subjects used for each unique task can be found in our github repo). Briefly, these criteria checked for clipping of waveforms (values clamped at 0 and 4095), unrealistic heart rates (mean heart rates below 30 bpm or above 97 bpm, or constant at 48 bpm (Xifra-Porxas et al., 2021), and missing waveforms. A subset of fMRI scans for each task, consisting of only those scans whose physiological data were labeled as ‘clean’ by the above criteria, was included in this study. For detailed information about task fMRI scans, please see Supplementary Table S2.

#### 2.1.4 Dataset 3

A different, in-house dataset was used as an external validation dataset. All subjects provided written informed consent, and human subjects protocols were approved by the Institutional Review Boards of the National Institutes of Health and Vanderbilt University. fMRI data were acquired with a multi-echo, gradient-echo EPI sequence, with the following parameters: TR = 2.1 s, duration of 24.5 mins, voxel size of 3 mm isotropic, echo times of [13.0, 29.4, 47.5 ms], flip angle = 75 deg, 30 axial slices (for detailed acquisition information, see Goodale et al., 2021). The scans were acquired under five different conditions: cued deep breaths, eyes-open auditory task, eyes-closed auditory task, passive eyes-closed auditory task (i.e., no button press), and eyes-closed rest (no stimuli presented). Each subject had scans from one or more (but not all) of these conditions. In the auditory tasks, auditory tones were delivered at long inter-stimulus intervals, and subjects were instructed to press a button as quickly as possible. Among scans that had undergone visual inspection to ensure they had clean physiological data, we selected a random subset of scans for the current tests. This yielded a total of 23 scans (drawn from 11 subjects).

### 2.2 Preprocessing

#### 2.2.1 fMRI

Both HCP-YA resting-state and task fMRI scans had undergone the HCP minimal preprocessing pipeline (Glasser et al., 2013). Beyond this, we applied linear and quadratic detrending to remove slow scanner drifts. This was followed by band-pass filtering within the frequency range of (0.01–0.15 Hz) and temporal downsampling by a factor of 2 (which does not result in further information loss, since the Nyquist criterion is satisfied). This step was performed in order to make HCP data comparable to more conventional fMRI acquisitions, in which the temporal sampling rate is typically slower than 1 s. Hence, the temporal downsampling is a strategic decision to enhance the practical utility of our models. By training on the longer TR data, *DeepPhysioRecon* becomes compatible with more widely available fMRI acquisitions, and facilitates retrospective analysis of datasets obtained with conventional acquisition parameters. The HCP minimal preprocessing pipeline included motion coregistration, and while an additional step of regressing out head motion parameters is an integral part of many fMRI preprocessing pipelines, we did not regress out head motion parameters when training our models since they may carry predictive information about physiological fluctuations. This decision was based on indications that apparent head motions can be introduced by respiration (Power et al., 2019), and our previous findings indicated that retaining head motion slightly improved the reconstruction of the respiration variation signal (Salas et al., 2021). Therefore, head motion was retained in the HCP resting-state dataset, which was used in model training, to preserve information relevant to predicting RV and HR. The in-house dataset, which was used exclusively for external validation of these trained models, was preprocessed according to a multi-echo ICA pipeline described in Goodale et al. (2021), as these scans were collected using multi-echo fMRI sequences. While similar to the other datasets, head motion parameters were not regressed out of this dataset, we note that the multi-echo ICA may effectively suppress residual head motion fluctuations (Kundu et al., 2013). All fMRI scans were spatially aligned to the MNI152 template.

#### 2.2.2 Physiological recordings

From the pulse oximetry signal, heart rate (HR) was extracted as the inverse of the mean inter-beat-interval in sliding windows of 6 s centered at each fMRI time frame (TR). Likewise, the respiration variation (RV) signal was calculated as the temporal standard deviation of the raw respiration waveform in a window of 6 s centered at each TR (Chang et al., 2009). Both RV and HR were then band-pass filtered (0.01–0.15 Hz) and were resampled to temporal resolution of 1.44 s, in a manner identical to the HCP fMRI data, the fMRI data that is used to train the models.

#### 2.2.3 Normalization

fMRI signals and respiration belt data were both acquired with arbitrary units and may carry scan- and subject-specific amplitude differences. Unless stated otherwise, all time-series signals, including heart rate, were temporally normalized to zero mean and unit variance.

#### 2.2.4 Dimensionality reduction

When training a neural network on voxelwise, 4D whole-brain data, downsampling or patch/window-based implementations are typically required due to GPU memory limitations. Extracting time courses that are averaged within functional or anatomically defined regions of interest (ROIs) enables computationally efficient modeling. The use of ROI time courses also renders the approach less sensitive to the spatial resolution of the acquired fMRI data. Therefore, here we carried out dimensionality reduction by parcellating the brain into regions of interest based on four published atlases. Of note, our learning framework can flexibly accommodate any set of atlas regions for dimensionality reduction. The four published atlases used here were derived from multiple imaging modalities: a cerebral cortex atlas that was derived from rs-fMRI data provided 400 cortical regions embedded within 7 larger functional networks (Schaefer et al., 2018); the Pandora TractSeg white matter atlas, which was derived from diffusion MRI data and included 72 uni-/bi-lateral white matter regions (Hansen et al., 2020); the Melbourne subcortex atlas, which provides a multi-modal segregation of 16 subcortical regions (Tian et al., 2020); and an ascending arousal network (AAN) atlas that includes 9 regions located in the brainstem (Edlow et al., 2012). Dimensionality and noise were reduced by extracting the mean fMRI time series from all voxels within each ROI.

All four atlases were already registered to MNI152 space, and when necessary, were also resampled to 2 mm isotropic voxels to match the resolution of the preprocessed fMRI scans. While the Pandora white matter atlases (Hansen et al., 2020) include various options, a probabilistic atlas created using TractSeg method (Wasserthal et al., 2018) on the HCP-YA cohort was utilized in this study. We thresholded the probabilistic atlas (at 95%) to exclude voxels with lower confidence and minimize the overlap between white matter and gray matter regions. The 7 Tesla HCP Scale I atlas from Melbourne subcortex atlases (Tian et al., 2020) was selected, with the assumption that 7T may provide better spatial precision and the Scale I granularity could support generalizability to external cohorts.

### 2.3 *DeepPhysioRecon* framework

We developed a generalizable deep learning framework to estimate RV and HR from fMRI data. We hypothesized that RV and HR share information that can foster their mutual learning, such that joint learning of RV and HR may enhance model accuracy and generalizability.

#### 2.3.1 Network architecture

**Fig. 1.**
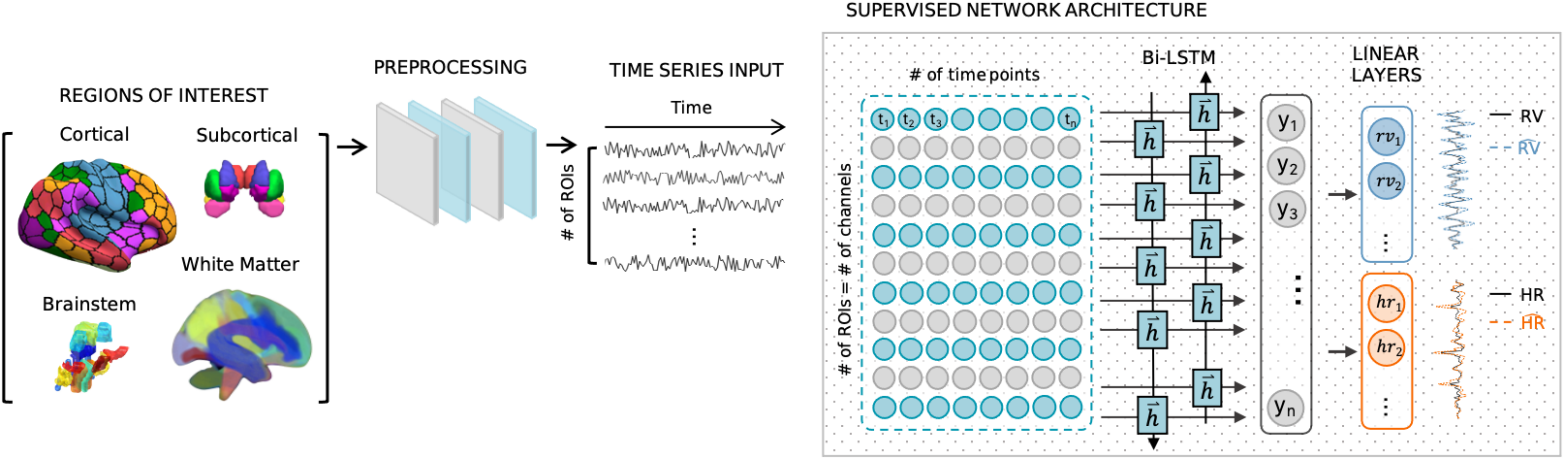
*DeepPhysioRecon* Pipeline. The pipeline for estimating respiration volume (RV) and heart rate (HR) signals from fMRI time-series dynamics is shown. Regions of interest are defined using four published atlases that had been constructed from different imaging modalities, comprising areas in cerebral cortex, white matter, subcortex, and the ascending arousal network. ROI time-series signals are extracted from the fMRI volumes, detrended, bandpass filtered and downsampled. The preprocessed signals are provided to a candidate network as input channels. A bidirectional LSTM network architecture is adapted for joint estimation. The output of linear layers are RV and HR signals.

Deep neural networks have shown remarkable success for image and time-series data (Sharma & Singh, 2017), including in the field of fMRI. The success of these networks stems from their ability to find a non-linear representation of the data and to make meaningful connections between spatial and temporal information implicit in the data. The proposed framework consists of a bidirectional LSTM (bi-LSTM) network followed by dropout and two linear layers. Long short-term memory networks (LSTM) were selected as a candidate approach based on their capacity to automatically learn the temporal dependencies present in time series and their capability of operating on data of varying lengths (Van Houdt et al., 2020). Linear layers are commonly used for inferring an objective-specific feature space, and here they were used to infer unique RV and HR estimates from LSTM hidden units. The network architecture is illustrated in Figure 1.

#### 2.3.2 Implementation details

To reduce computational demands and improve signal-to-noise ratio, we employ atlases for dimensionality reduction. The input to the networks consist of ROI time-series signals provided as different channels (# of channels = # of ROIs). The bi-LSTM network is followed by a dropout layer and is connected to two linear layers, which output the estimated RV and HR time series. These estimated signals are the same length as the corresponding input ROI time series.

We conducted a grid search to select hyperparameters for the resting-state models. The size of hidden states was selected from values of [32, 64, 512, 1000, 1024, 2000, 2048], batch size from values of [1, 2, 8, 16, 32, 64], for dropout rates of [0.1, 0.3, 0.5, 0.6], for learning rates from [1.0e-2, 1.0e-3, 1.0e-4, 1.0e-5], and for the decaying learning rate with decay rate of [0.01, 0.1, 0.2, 0.5].

All models were then trained with the following, empirically chosen hyperparameters: a hidden state (h) size 2000 (i.e., depth), a batch size of 16, dropout rate 0.3 and were trained with decaying learning rate (lr) of 1.0e-3 with patience 2 and decay rate of 0.5, saving only the best models according to validation performance. The models were trained using ADAM optimizer with default parameters. The experiments were performed on an NVIDIA RTX 2080Ti GPU. Programs were implemented with Python using the Pytorch deep learning library.

#### 2.3.3 Model training and evaluation

##### 2.3.3.1 Resting state (rs-) models

When training and evaluating the model solely on the HCP-YA data (Datasets: Dataset 1), we used five-fold CV. This involved using 80% of the data for training, which was further divided into 68% for actual training and 12% for validation, while the remaining 20% was set aside for testing each fold. A subset of 375 subjects (1500 scans) were used for training by rotating the partitions (Supplementary Fig. S8; left), and the resulting performance (pooled over the 5 testing partitions) is reported. In addition, to evaluate the generalizability across conditions (task vs. resting state) and acquisition parameters, rs-models were tested on HCP-YA task (Dataset 2) and in-house (Dataset 3) datasets. Since the five-fold CV on the resting-state fMRI data resulted in 5 different models, one of these models was randomly selected to be applied to the task and in-house datasets (Supplementary Fig. S8; right). For detailed information about number of scans used for training and testing, please see Supplementary Table S3.

##### 2.3.3.2 Training task (t-fMRI) model

t-fMRI model is trained using HCP-YA task fMRI dataset Dataset 2). More specifically, models were trained using 4 tasks (working memory, social cognition, emotional processing, and gambling tasks), and assessed using the remaining 3 tasks (relational processing, motor and language tasks). Of note, the subjects that were included in the training t-fMRI model were excluded from testing cohorts (Supplementary Fig. S9; left), in order to assess generalizability across different subjects. In addition, to further evaluate the generalizability across conditions (task vs. resting state) and acquisition parameters, t-models were also tested on HCP-YA resting-state (Dataset 1) and in-house (Dataset 3) datasets (Supplementary Fig. S9; right). For detailed information about the number of scans used for training and testing, please see Supplementary Table S4.

### 2.4 Impact on fMRI signals and network connectivity

#### 2.4.1 Percent variance explained

To assess the degree to which our reconstructed RV and HR signals could account for fMRI signal fluctuations across the brain, we examined the percentage of temporal variance explained in each fMRI voxel signal by the reconstructed RV and HR signals. The predicted time courses were first convolved with a previously determined transfer function (for RV, respiration response function (Birn et al., 2008); for HR, cardiac response function (Chang et al., 2009)) that captures the forward mapping between physio and fMRI fluctuations, as well as their time and dispersion derivatives (Chen et al., 2020) to allow for small deviations in latency and shape from the canonical model (Henson et al., 2002). The percent variance explained was defined as the fraction by which a voxel’s original temporal variance would be reduced after projecting out (via ordinary least squares) a linear combination of the aforementioned regressors, and multiplying by 100. For each scan, the RV and HR predictions used in this analysis were obtained from cross-validated rs-models, where the model applied to derive a subject’s predicted RV/HR signals was one that had excluded that subject from the training. This analysis used the HCP-YA resting-state data that has been bandpass filtered and downsampled by a factor of 2 (see Section 2.2 for details). The percent variance maps calculated from individual scans were then averaged to get the population-level mapping of the RV and HR effects.

#### 2.4.2 Connectivity analysis

For both the ROI-based and seed-based connectivity analyses, we used resting-state scans that have undergone the HCP ICA-FIX preprocessing pipeline (Griffanti et al., 2014). The rationale behind using ICA-FIX data for this analysis is to show the utility of physiological signals even beyond ICA-FIX denoising, as substantial RV and HR effects may remain (Kassinopoulos & Mitsis, 2019). Beyond ICA-FIX, we applied linear and quadratic detrending to remove slow scanner drifts. This was followed by band-pass filtering within the low-frequency range of (0.01–0.15 Hz) and temporal downsampling by a factor of 2.

##### 2.4.2.1 ROI-based

To assess the impact of the physiological signal of interest on regional correlations, we pursued a functional connectivity analysis. Functional connectivity commonly refers to similarities in brain activity signals between regions, and is calculated as the pairwise (Pearson) correlation of ROI time series and represented as a symmetrical matrix. We assessed the pairwise functional connectivity between all 497 ROIs drawn from cortical, white matter, subcortical, and ascending arousal network atlases (Section 2.2.4) both before and after projecting out the estimated physiological subspace using the same basis functions described above (see Section 2.4.1), as well as 6 rigid-body head motion parameters and their derivatives. The Pearson correlation between each pair of ROIs were calculated and used to construct 497 x 497 symmetrical connectivity matrices where each element represents temporal similarity score between two ROIs.

##### 2.4.2.2 Seed-based

Seed-based analysis is one of the most common ways to explore functional connectivity within the brain (Van Den Heuvel & Pol, 2010). In a seed-based correlation analysis, connectivity is calculated as the correlation between the time course of a selected reference (“seed”) region to all other voxels in the brain. The resulting connectivity map represents the Pearson correlation scores for each voxel, indicating how well each voxel’s time series correlates with the time series of the seed. In this analysis, we pursued seed-based connectivity with respect to a seed region in the default mode network (DMN). For comparison, these analyses were repeated after projecting out the measured and predicted RV and HR waveforms, using the basis sets and derivatives described above (see Section 2.4.1), as well as six rigid-body head motion parameters and their derivatives.

## 3 Results

In Section 3.1, we first show the agreement between measured and decoded RV and HR signals. In Section 3.2, we demonstrate how *DeepPhysioRecon* can enable investigating these low-frequency physiological effects in the absence of measured respiration and cardiac data. Next in Section 3.3, we assess model generalizability and performance across datasets and experimental conditions. Sections 3.4 and 3.5 explore the interpretability of our learning framework. Finally, in Section 3.6 we investigate *DeepPhysioRecon* as a potential tool for denoising RV and HR measures derived from corrupted physiological recordings.

### 3.1 Decoding low-frequency peripheral physiological signals from resting state data

**Fig. 2.**
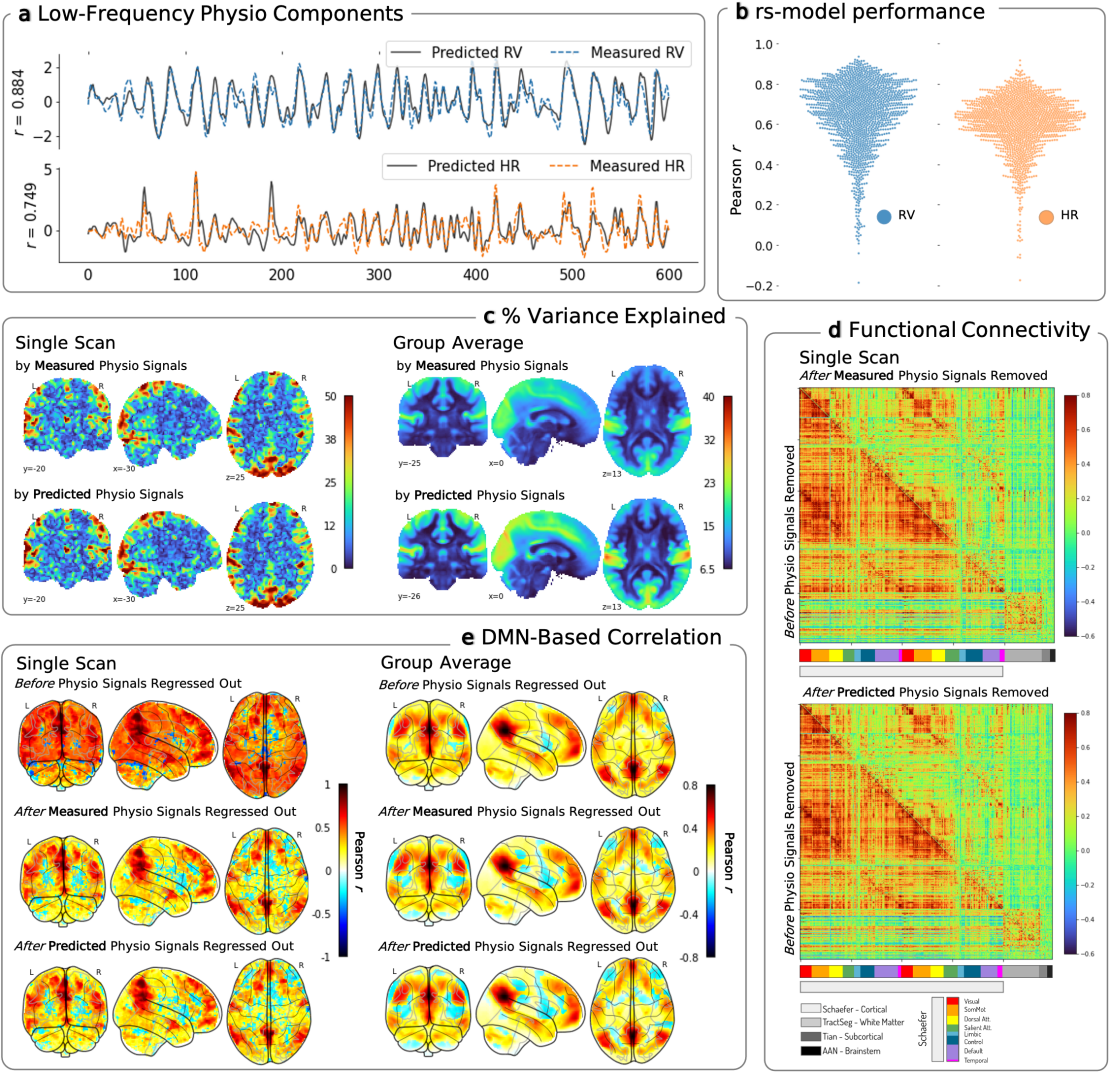
Resting-state model performance on withheld test data. (a) Low-frequency physiological signals predicted by our model are overlaid on the measured signals for one example scan. Accuracy is measured using Pearson correlation coefficient between measured and predicted signals. (b) Models are trained and evaluated with resting-state data under a five-fold CV paradigm. Each marker represents the Pearson correlation (r) scores between measured and predicted signals for respiration variation (RV – shown in blue) and heart rate (HR – shown in orange), pooled across the withheld resting-state test sets. Single scan and group effects observed on resting-state fMRI data. (c) Percent (%) variance explained maps shown for selected slices, indicate the percentage variance explained at each brain voxel by the measured and predicted physiological signals, for one example scan and averaged across the subject group. (d) Functional connectivity matrices highlight the change in ROI-to-ROI correlation after the indicated signals were removed from the ROI time-series data for one example scan. (e) DMN-based correlation maps (seed coordinates (2,-58,30) in MNI152 template space) at the voxel level show the seed-based correlation across the brain regions before and after the measured and predicted physiological signals are regressed out from the fMRI data, again for one example scan and averaged across the subject group.

We first demonstrated the applicability of our joint learning approach using the resting-state dataset. Training was performed on 375 subjects, each scanned 4 times, totaling 1500 scans. The resulting models are referred to as *rs-models*. To ensure the accuracy and robustness of these models, five-fold CV was employed (see Section 2.3.3). Figure 2a shows the results for one example scan, where decoded signals aligned with the measured (ground truth) signals with a Pearson correlation of r = 0.884 for RV and r = 0.749 for HR. The resulting model accuracy for all folds shown in Figure 2b. The proposed framework reconstructs RV and HR signals on test cohorts with high agreement against those calculated from the measured physiological data, with a median r ∼0.679 for RV, and median r ∼0.625 for HR. To note, the five CV folds produced models with highly similar performance on the test partitions (see Supplementary Fig. S6). The intrinsic autocorrelation of the RV and HR time-courses potentially introduces a positive bias in the computation of Pearson correlation. To establish a baseline, we mismatched subjects and calculated the mean correlations between the measured and predicted RV (or HR) drawn from distinct subjects. In this shuffled data, the mean Pearson’s r between measured and predicted RV was 0.00307, and -0.00224 for HR. Further, to assess whether the joint learning method was able to distinguish HR and RV variations, we also report the mean correlation between the measured RV and HR is 0.275 ± 0.19, while that between the predicted RV and HR is 0.456 ± 0.16.

### 3.2 Impact on fMRI signals and network connectivity

We next demonstrate how these decoded physiological signals can—in the absence of physiological recordings—be used in widely conducted fMRI analyses, and compare the results against analyses conducted using measured (“ground truth”) physiological signals. Figure 2 provides examples of the impact of decoding RV and HR at the single-scan and the group levels.

To gauge the influence of RV and HR on fMRI, we first show the proportion of temporal variance explained by a linear combination of RV and HR effects in the fMRI signal at each voxel, across the whole brain. For the example subject in (Fig. 2c), the variance explained by the measured and predicted RV/HR signals exhibits similar patterns, and the magnitude reaches approximately 50%; that is, there are parts of the brain where half of the fMRI signal fluctuation is accounted for by RV/HR. When averaged across the entire set of 1500 scans, the variance explained by RV and HR signals across the brain ranged from 6.5 to 33% (Fig. 2c), and also show similar spatial distributions. See Supplementary Figure S2 for the regional distribution of variance explained for each ROI.

We next assess the impact of these decoded physiological signals on functional connectivity analysis. Often, physiological signals are removed from fMRI data to control the influence of these systemic effects on inferences of functional connectivity. In this study, if *DeepPhysioRecon* proves successful, eliminating the measured (ground truth) and predicted (decoded) RV and HR signals should similarly influence functional connectivity.

Therefore, we assess the functional connectivity between all 497 ROIs drawn from the aforementioned cortical, white matter, subcortical, and ascending arousal network atlases, both before and after projecting out the estimated physiological subspace. Further details of the projection are provided in Section 2.4.1. The Pearson correlation between signals from each pair of ROIs is used to construct a 497 x 497 symmetrical connectivity matrix. As a complementary analysis, we also map the whole-brain, voxel-wise connectivity with respect to a reference (“seed”) region in the default mode network (DMN), both before and after removing the estimated physiological subspace. We observe that both the ROI-based (Fig. 2d - Single Scan) and seed-based (Fig. 2e - Single Scan) correlations are altered with the removal of physiological signals, and that comparable results are obtained using the measured and decoded physiological waveforms, as shown for an example scan. When averaged across all 1500 scans, regressing out the predicted and measured physiological signals had a more mild effect overall but also exerted similar effects on the DMN, enhancing negative correlations while sustaining positive correlations (Fig. 2e - Group Average), as expected (Chai et al., 2012; Chang & Glover, 2009). Similar effects of measured and predicted physiological signals were also obtained at the group level (see Supplementary Fig. S4 for ROI-to-ROI functional connectivity and Supplementary Fig. S5 for seed-based connectivity).

### 3.3 Generalizability across tasks and acquisitions

The above results indicate that RV and HR can be decoded from resting-state fMRI data. To what degree do these models directly generalize to other fMRI paradigms or acquisition parameters? To test this question, we applied a randomly selected model from the five-fold CV on resting-state data, without any additional training or fine-tuning, to data acquired during seven different task paradigms from the HCP-YA dataset. Further, as an even stronger test of model generalizability, we also evaluated the performance of the HCP-YA resting-state model, again without any additional training, on an external dataset that was acquired on a different scanner and with different acquisition parameters, including temporal resolution (results from models built from each of the five resting-state folds are reported in Supplementary Figs. S6 and S7). Further details about the datasets and preprocessing are described in Section 2.1 and Section 2.2.

**Fig. 3.**
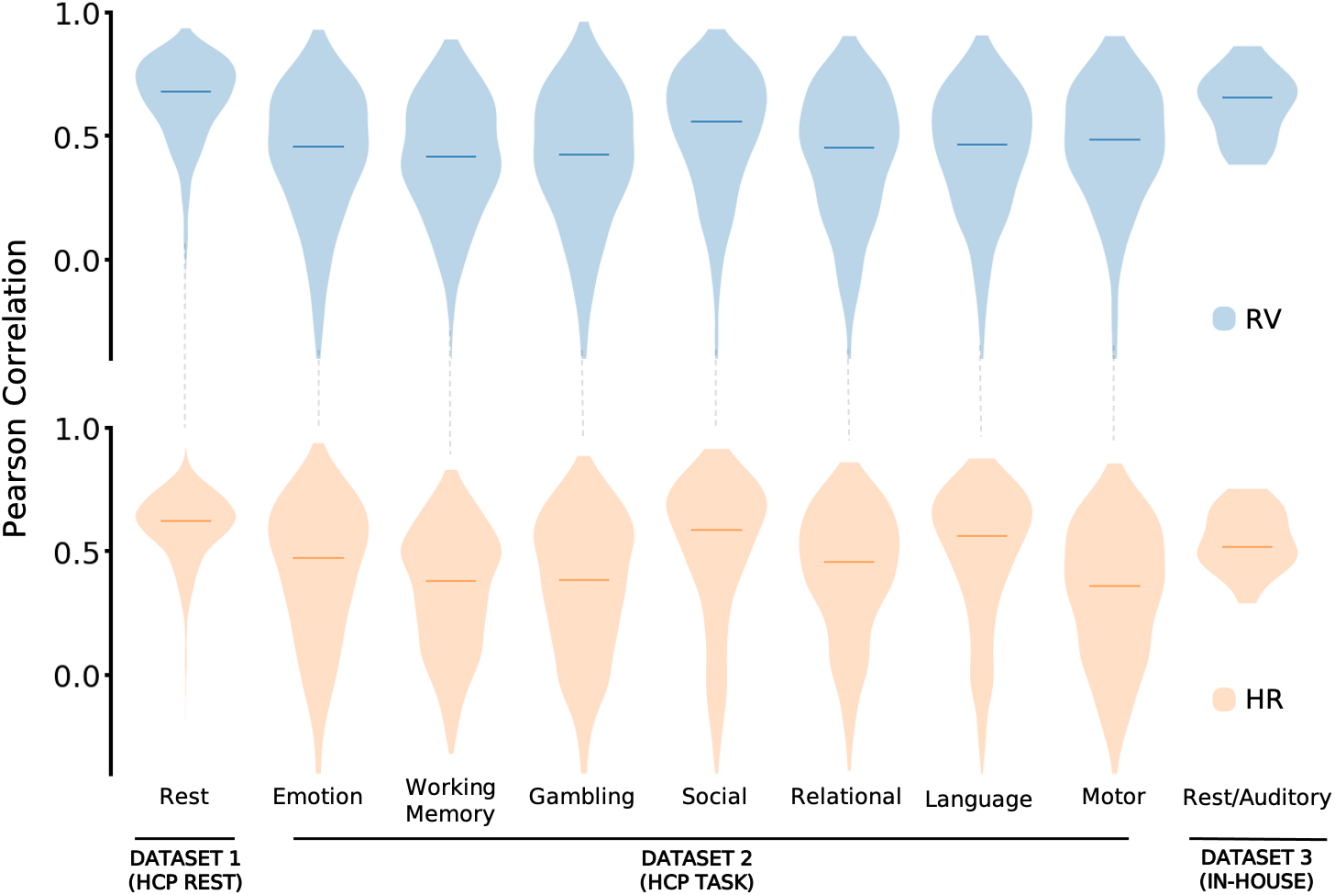
Generalizability of rs-model to scans acquired under different experimental conditions and acquisition parameters. From the five models trained on resting-state data under the five-fold CV paradigm, a model is randomly selected for this assessment. Seven tasks from Dataset 2 (HCP: emotion, working memory, gambling, social, relational, language and motor), and Dataset 3 (in-house: rest/auditory) were used to assess generalization of the model. Each plot represents the aggregated Pearson correlation scores between measured and predicted signals for respiration variation (RV – shown in blue) and heart rate (HR – shown in orange) in the withheld test set. Median r is indicated by the horizontal line.

The results (see Fig. 3) suggest that for the held-out datasets, the model trained on the HCP-YA resting-state data captures a transferable feature space. The agreement between measured and predicted signals for RV was high in the in-house dataset (median r > 0.6), and moderate for all HCP-YA tasks. For HR, the performance was also high for the in-house dataset. Moreover, HR had notably high performance on the HCP-YA social and language tasks (median r ∼0.6 ), and a moderate agreement for the other HCP-YA tasks.

**Fig. 4.**
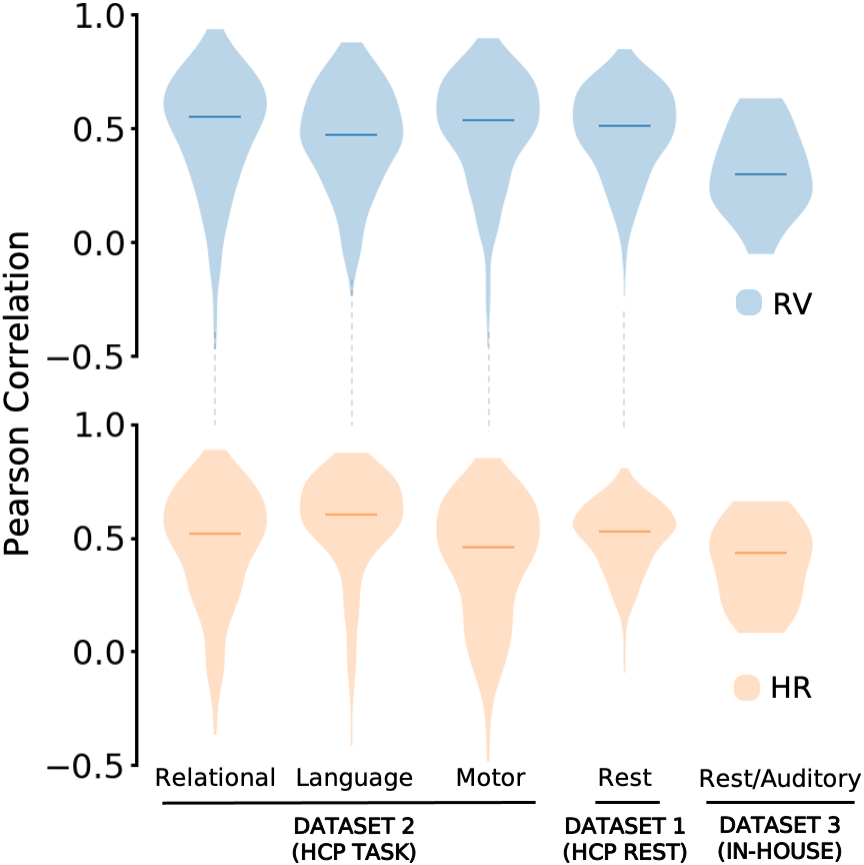
Generalizability of t-model on test cohorts acquired with different task conditions and acquisition parameters. Three tasks from Dataset 2 (HCP: relational, language and motor), Dataset 1 (HCP: resting-state) and Dataset 3 (in-house: rest/auditory) were used to assess generalization of a model trained on data consisting of 4 other task conditions. Each plot represents the aggregated Pearson correlation scores between measured and predicted signals for all scans in a set. Median r is indicated by the horizontal line.

Finally, we asked whether training models on task data could improve the performance on datasets involving tasks that are different from those on which the model is trained. This question also probes whether the relationship between fMRI and physiology is largely independent of task condition. To investigate this question, we preserve the same framework used for the rs-model (including hyperparameter values and model architecture) and train a new model, which we refer to as the t-model, using only four of the seven tasks (working memory, social cognition, emotion processing, and gambling tasks). The t-model is then deployed to test generalizability on the three remaining tasks (relational processing, language, motor tasks), as well as on the HCP-YA resting-state scans and the in-house dataset. The results show (see Fig. 4) that overall, the t-model is able to learn both RV and HR with moderate agreement against ground truth (median r ∼0.5 ). When tested on the HCP-YA tasks, the t-model showed an improvement in performance compared to the rs-model (Supplementary Table S5).

### 3.4 Spatially constraining what the network sees

**Fig. 5.**
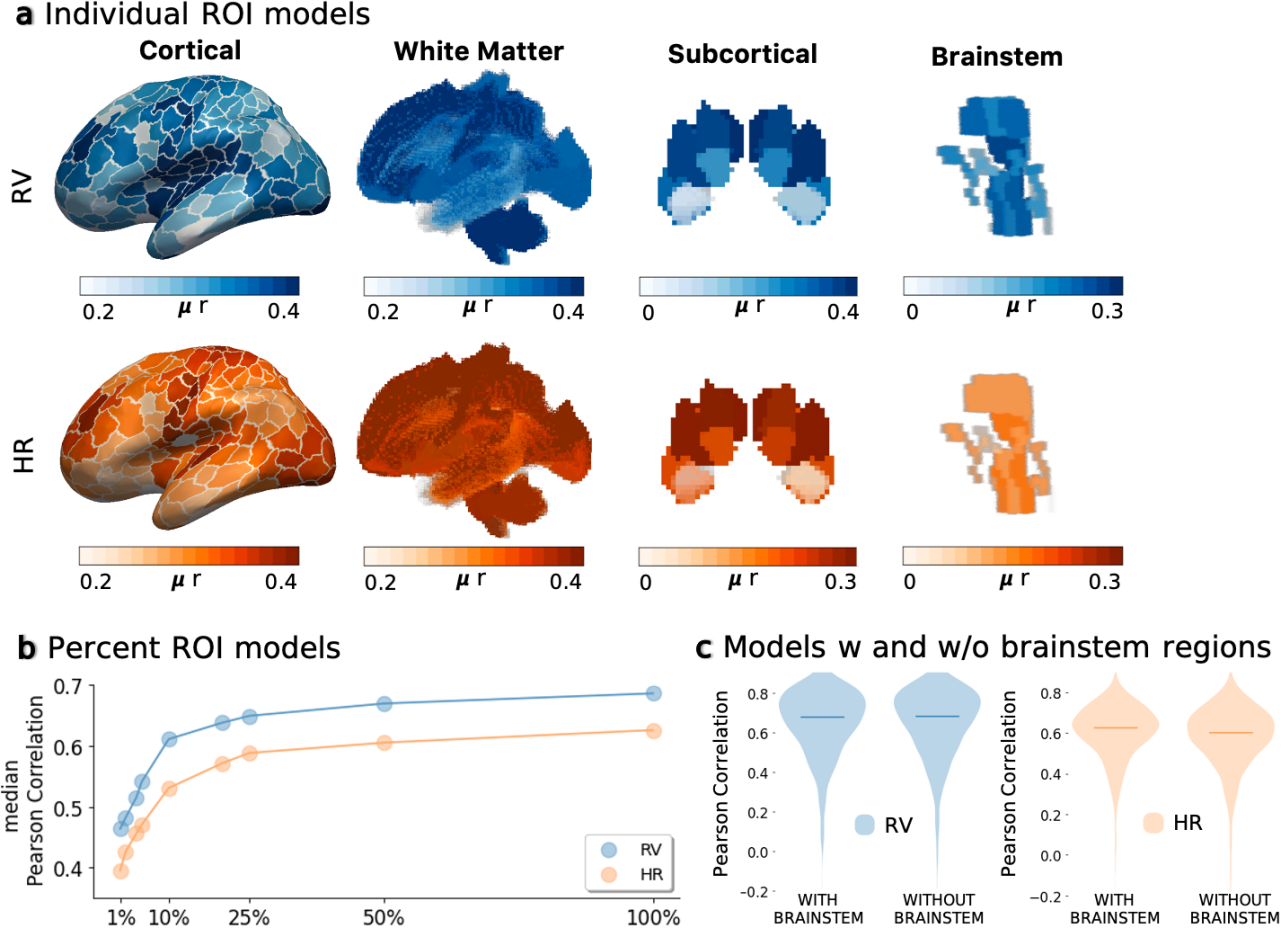
(a) Individual ROI models. Using resting-state data, models were trained separately for each individual ROI. Predictiveness of a given ROI was assessed using Pearson correlation between measured and predicted signals. Results were visualized by projecting the mean r onto (left to right) cerebral cortex (400 ROIs), white matter bundle regions (72 ROIs), subcortical regions (16 ROIs) and ascending arousal network (9 ROIs). (b) Percent ROI models. A set of models are trained with an increasing number of ROIs. Starting from the top 1% of the total ROIs, rank-ordered by results of the individual ROI analysis, models are trained using successively increasing numbers of ROIs, up to 100% of ROIs. (c) Model comparison without brainstem and cerebellar regions. A model is trained using 477 ROIs, excluding regions that spatially overlap with brainstem and cerebellum. Each plot represents the Pearson correlation scores between measured and predicted signals for all scans in the unseen test data.

Our learning framework can flexibly accommodate any set of atlas regions for dimensionality reduction. Although we chose a large number of brain regions when fitting the original model in order to maximize brain coverage, we hypothesized that certain brain regions convey more physiological information than others. For example, regions near major blood vessels may be more strongly influenced by physiology, and thus provide greater predictive value. To investigate this hypothesis, three linked experiments are designed. In the first, we use only one input ROI times-series signal at a time, generating what we will refer to as individual ROI models. These models are trained in a manner identical to that of the original (multi-ROI) model. The model performance for each scan is assessed using Pearson correlation between the predicted and measured physiological signals, and an average score for the entire test cohort is noted (see Section 2.3.3). Figure 5a shows these average scores mapped onto their corresponding regions for all 497 regions, representing the predictive ability of each individual ROI. The best performance of individual ROI models reaches r ∼0.5 (note that colorbar limits were set to a smaller range to maximize image contrast).

Next, we assess whether the number of regions included in training affected the model performance. Here, models are trained using successively increasing numbers of ROIs (from 1% to 100%), rank-ordered by the results of the individual ROI analysis. These models will be referred to as percent ROI models. While the improvement in model performance continuously increased with the number of included ROIs (Fig. 5b, i.e. for RV median Pearson r ∼0.45 and r ∼0.65 respectively with 1% and 25% of ROIs), the rate of increase slows after the model includes approximately 25% of the top ROIs, and reaches median Pearson r ∼0.7 with 100% of the regions.

Given that brainstem and cerebellar regions are often absent from fMRI acquisitions, and yet are implicated in cardiovascular regulation, the last experiment assesses the performance when using all regions except those that spatially overlap with brainstem and cerebellum. The results indicate that excluding brainstem and cerebellar regions resulted in comparable performance and did not hamper the prediction accuracy (Fig. 5c).

### 3.5 Factors driving model performance

**Fig. 6.**
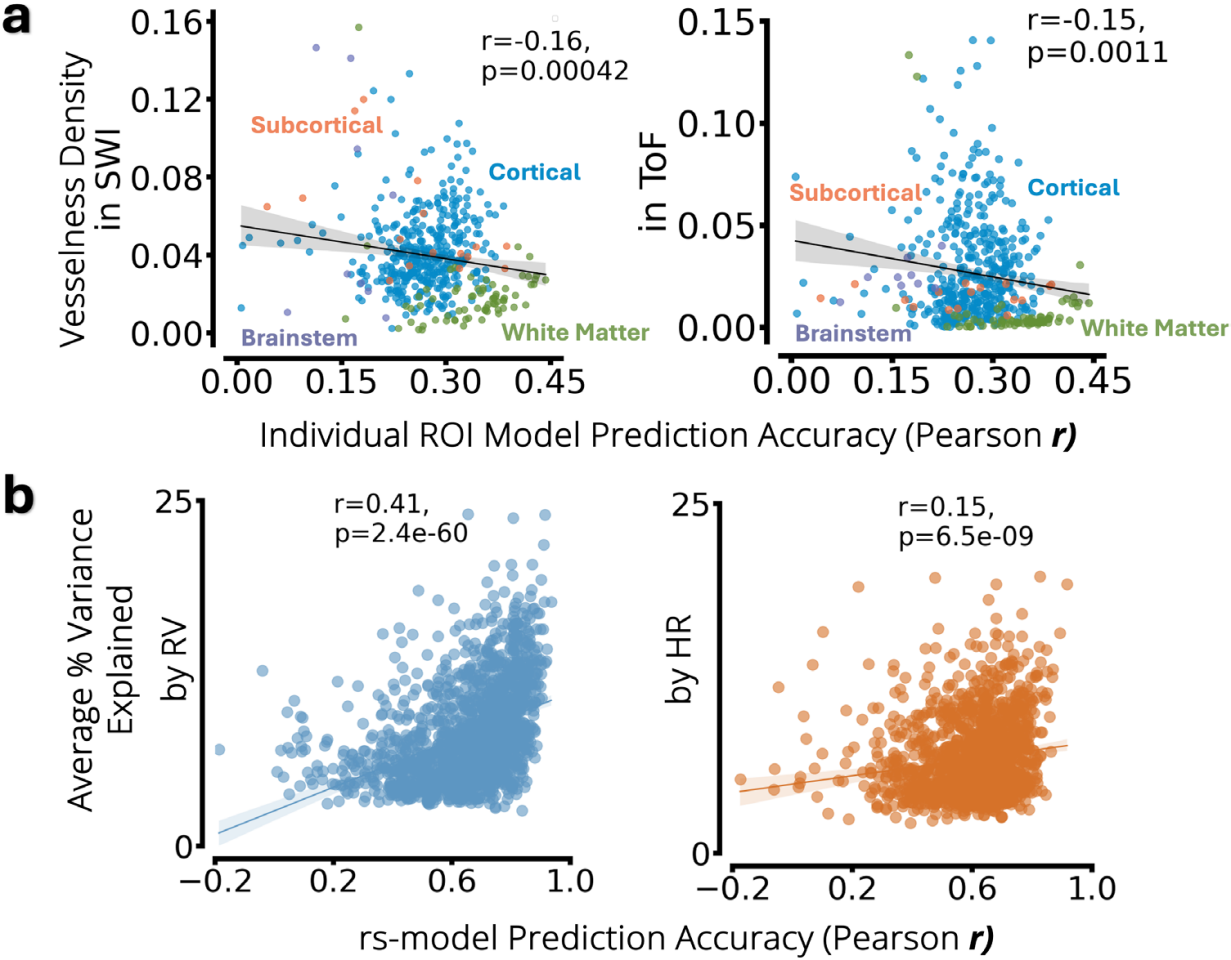
(a) Vessel density as a factor that may underlie regional prediction accuracy. In each plot, the x-axis represents model accuracy for each individual-ROI model. The y-axis represents the average vessel density for Susceptibility Weighting Imaging (SWI; left) and Time-of-Flight (ToF; right) measures, averaged across all voxels within each ROI. (b) Percent variance accounted for by physiology in fMRI data, as a factor that may underlie model prediction accuracy. In each plot, the x-axis represents cross-validated model accuracy calculated using Pearson correlation scores between measured and predicted signals for respiration variation (RV – shown in blue) and heart rate (HR – shown in orange) in the withheld resting-state test set. The y-axis represents the percentage of temporal variance explained in the fMRI data, averaged across all brain regions, by the respective (measured) physiological signal.

We further investigated the various factors that may drive or affect model performance. For this, we first examine whether the results are driven by BOLD signal in regions near blood vessels. To investigate this relationship between the “vesselness” of a region and its relative predictive ability, we carried out a linear regression analysis between Time of Flight (ToF) and Susceptibility Weighting Imaging (SWI) measurements of vessel density (Bernier et al., 2018) in an ROI and the prediction accuracy of the corresponding individual ROI models. The results are shown in Figure 6a. Interestingly, the vessel density metric is negatively associated with predictive value (r = -0.15 p-value = 0.0011 for TOF, r = -0.16 p-value = 0.00042 for SWI), showing that the higher the vessel density, the less accurate the model predictions from the fMRI signal within that region. Further analysis indicated that if white matter regions were excluded from the analysis, no significant correlation was observed (r = 0.02, p-value = 0.74 for TOF, r = 0.01, p-value = 0.87 for SWI), suggesting that white-matter regions—with their low vascular density yet high predictive performance—are driving the negative correlation.

We also hypothesized that the magnitude of RV and HR fluctuations themselves relates to the magnitude of their influence on brain hemodynamics, and therefore could potentially impact the ability to predict physiological measures from an fMRI scan. To test this hypothesis, we conducted a linear regression analysis. Specifically, we examined the relationship between the amount of fMRI temporal variance explained in a scan (which represents physiologically induced BOLD variations) averaged across all brain voxels, and the accuracy of our model in predicting RV/HR. The results from this analysis, indeed, support our hypothesis, as we found a positive correlation between the two (Fig. 6b). This suggests that the degree of physiologically induced BOLD variation during a scan directly influences the ability of a model to accurately predict RV/HR from the scan.

As shown in prior work (Salas et al., 2021), not regressing out head motion artifacts (translations and rotations) in the fMRI data improved the quality of the RV predictions in prior work. However, retaining head motion effects may not be helpful when estimating HR, which may be more closely linked with within-brain pulsatile motion than with bulk shifts. To test this possibility, we assessed the potential relationship between a subject’s overall motion during a scan and the performance of HR prediction. The results (see Supplementary Fig. S10) indicated a subject’s movement was not significantly correlated with the performance of HR predictions.

### 3.6 Potential for denoising physiological waveforms

**Fig. 7.**
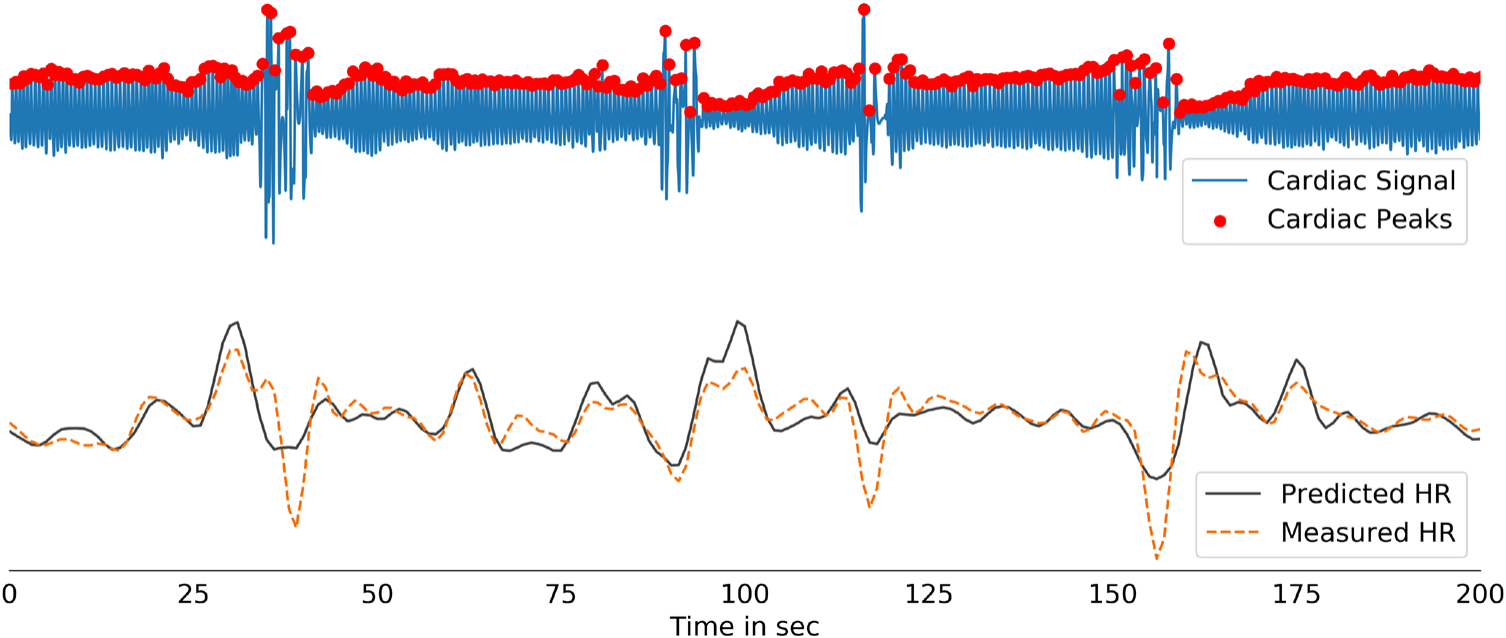
An example recording is shown. The transient artifacts in the HR waveform (bottom; orange) are likely due to motion in the raw PPG signal (top). *DeepPhysioRecon* may help to ‘fix’ noisy physiological recordings, as indicated by the predicted HR signal (bottom; black).

**Fig. 8.**
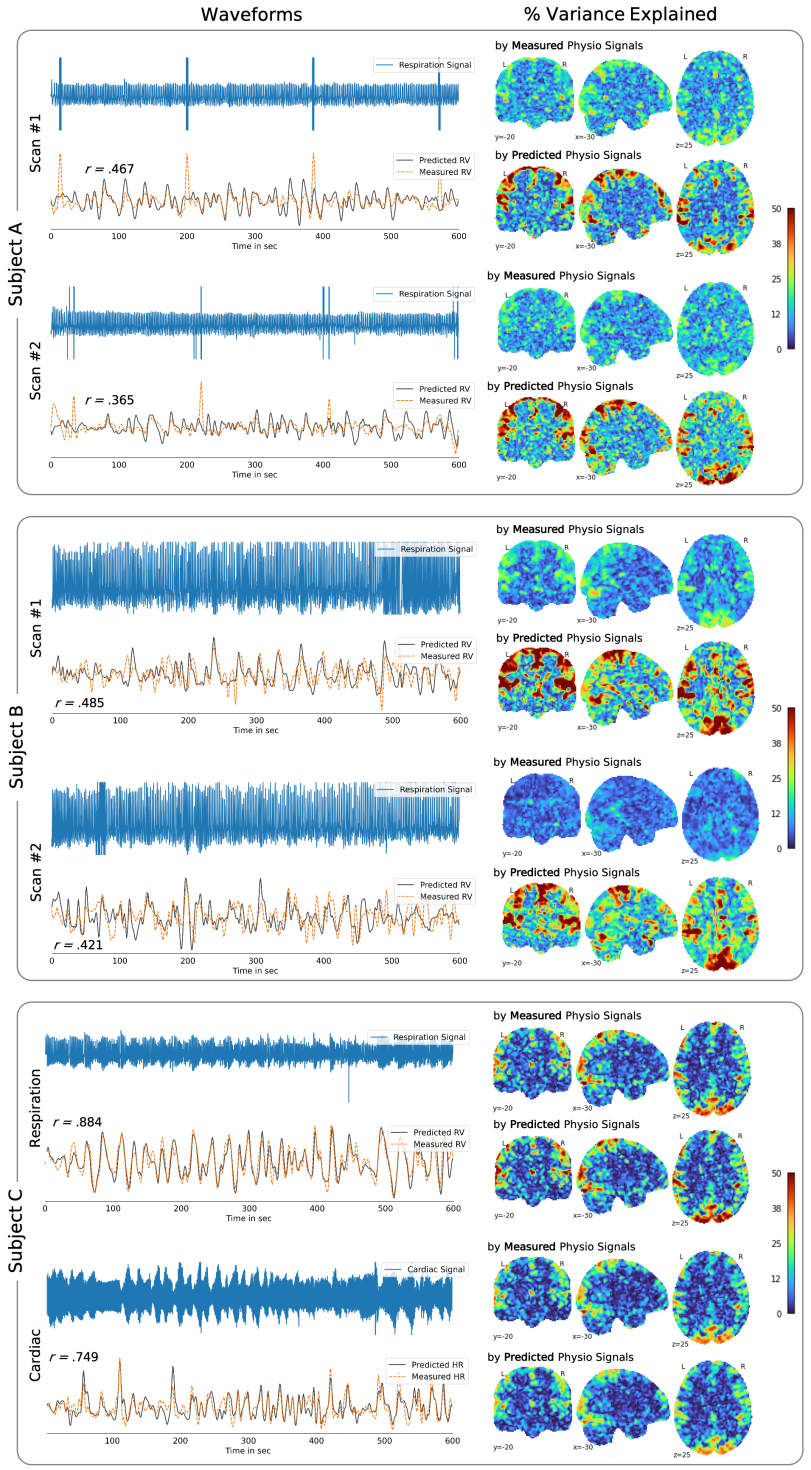
Low correlation between measured (‘ground truth’) and predicted RV signals may not accurately reflect the quality of reconstruction. In the selected examples, artifacts are observed in the raw time-series signals. For example, data from Subject A is shown to have periodic artifacts likely related to the equipment, and data from Subject B exhibits clipping artifacts. These are then carried over to the derived low-frequency RV and HR waveforms. We observe that the predicted waveforms appear to mitigate some of these artifacts, suggesting that the model may be able to clean up physiological signal measures through this data-driven learning process. The percent variance explained maps indicate that the predicted RV signals accounted for a much larger proportion of the fMRI signal variations compared to the measured RV signals. On the contrary, in the example of Subject C, the respiration and cardiac recordings from the same scan (without any major artifacts) are shown. The variance explained maps indicate that measured and predicted RV signals accounted not only for a similar proportion of the fMRI signal variations but spatial distribution of these maps closely aligned.

Low correlation scores can emerge when there are artifacts in the measured physiological recordings, coloring the performance during testing and therefore necessitating close inspection. We speculate that the signals predicted from *DeepPhysioRecon* may have the potential to ‘fix’ some of the artifacts in the measured physiological data. In Figure 7, we provide one example in which possible motion-induced artifacts can be observed in the raw cardiac signal, whose effects propagate to the “ground truth” low-frequency HR signal. We observe that the predicted HR smooths over these artifacts, suggesting that the model may be able to clean up RV/HR measures through this data-driven learning process. Another observation is that in some cases wherein the predicted RV/HR signals exhibited low correlation with the ground-truth RV/HR signals, the predicted signals explained a larger variance in the fMRI data compared to the measured physiological signals. Two such examples are shown in Figure 8 (Subject A, Subject B). Notably, although having stronger magnitudes, the spatial maps associated with the predicted physiological signals retain subject-specific patterns that are also present in the maps associated with the measured physiological signals (see also Supplementary Fig. S3). These examples may also indicate that the model can generate physiological estimates that are cleaner than the recordings. In cases where the measured physiological signals appear to be clean and have high correlation with the predicted waveforms (Subject C), close correspondence is also seen in their effects on fMRI data.

## 4 Discussion

This work presents a framework for jointly inferring respiration and heart rate fluctuations directly from fMRI dynamics. The *DeepPhysioRecon* framework provides these two key low-frequency physiological signals (RV, HR) to datasets that fully or partially lack external physiological measures, or which lack measurements of sufficient quality. We demonstrate that the proposed models of RV and HR, trained on resting-state fMRI data, generalize across datasets with varying experimental conditions and significant acquisition differences. Consistent with the literature (Birn et al., 2006; Chang et al., 2009; Kassinopoulos & Mitsis, 2019; Power et al., 2017), we also find that RV and HR can, in certain scans, account for large amounts of temporal variance in fMRI signals. By including the predicted RV and HR signals in functional connectivity analyses, we also show that large-scale network maps are altered in agreement with the changes introduced by incorporating the measured RV and HR signals. The proposed models are tested not only in independent participants within each dataset, but also across datasets acquired with different experimental conditions, MR scanners, and protocols.

Models trained and tested on resting-state fMRI data succeeded in decoding RV and HR signals with high accuracy. These models were also found to generalize well between experimental conditions, different subjects, and even across significantly different acquisition parameters, including different temporal resolutions (TR = 0.72 s to 2.1 s). Figure 3 indicates that models that were directly applied to different fMRI conditions (i.e. resting-state models applied to task, and task models applied to resting-state or unseen task conditions), could infer information in a new set of experimental conditions. This ability to transfer models between fMRI conditions suggests that the relationship between fMRI and physiology could be largely brain state-invariant. However, as expected, models trained using task fMRI data exhibited improvements on decoding task data in comparison to those trained on resting-state data (Fig. 4; Supplementary Table S5), though the degree of performance improvement varied across tasks. Likewise, models trained on resting-state data performed better on held-out resting-state data than they did on task data.

One possible explanation for this increase in accuracy is that within a particular condition (task vs. rest), it is expected that the distribution (signal and/or noise) matches more closely. For the case of task, there may be task-related brain activity that shows up in more systematic ways both in the fMRI data and physiological recordings. Therefore, we can speculate that the model learns to isolate these patterns when trained with task data. Further, the success of rs-models to more accurately decode an external dataset with different acquisition parameters, compared to the t-model, may also relate to the conditions used in the external (in-house) dataset. The in-house dataset tasks involved only simple sensory stimulation (auditory tones) that were presented at long-inter-stimulus intervals (ISI) for some scans, or at very short ISI but continuously throughout the experiment for others. Both of these designs will have more contribution from spontaneous fluctuations, resembling the resting-state conditions.

Nonetheless, we found that even when training and testing on the respective conditions, model performance was higher on the rs-fMRI data than for task fMRI data. One explanation may be that the resting-state scans were longer than the task scans. It has been shown that increasing the scan duration may increase the reliability of fMRI connectivity estimates (Birn et al., 2014), so perhaps training models with long duration (∼15 min) scans (as in the HCP-YA resting-state data) may offer advantages over training with the shorter-duration (2-5 min) scans.

We suggest that depending on the goal of the study, different training datasets should be considered. If the goal is to create the data in its absence (i.e., to reconstruct physiological signals in a dataset that does not include any physiological measurements), a large cohort including variety of fMRI conditions and acquisition parameters may give a more robust model. Training models using the same task as the one for which prediction is needed can further increase accuracy, as the models may be trained using the same dataset and same condition. While this will reduce the generalizability of a model across conditions, it may allow for better condition-specific reconstruction as well as for ‘fixing’ corrupted data within a dataset (i.e., cases in which the dataset has physiological measurements but contains corrupted samples). However in the latter case, we would extra caution against overfitting to the data, which can be achieved in various ways. For instance, initializing the weights by a model pretrained with large-scale fMRI data across a broad set of conditions can provide a good starting point.

Our neural network architecture was designed to handle many unique challenges related to fMRI. First, bi-directional LSTM networks support varying-length input and multivariate output. This allows for learning on varying-length input signals and for predicting RV and HR simultaneously. Given that fluctuations in respiration and heart rate evoke delayed responses in the BOLD signal (i.e., the BOLD effects occur asynchronously and persist beyond the duration of the physiological response) (Birn et al., 2008; Chang et al., 2009), a neural network that takes past and future information into account—such as bi-LSTM—means that no hemodynamic lag needs to be explicitly considered. Further, the ability to integrate over multiple time-scales may offer benefits over fixed-windowed methods (Salas et al., 2021). Relatedly, the measured (ground-truth) RV and HR time-series were intentionally not convolved with any assumed impulse response function. This design choice was made to avoid the constraints of specific assumptions, including linearity and the shapes within a predetermined basis set. Instead, by directly reconstructing RV and HR time-series, our neural network effectively carries out a form of “deconvolution,” extracting low-frequency physiological signals from the fMRI data. In our subsequent analyses, however, we did assume a linear convolution relationship for the purpose of estimating the percent variance explained in fMRI data by the resulting deconvolved signals (Section 2.4.1).

Given that RV and HR are strongly coupled, we had hypothesized that learning the features of one (i.e. RV) could boost the accuracy of predicting the other (i.e. HR) even with a few learning instances. However, experiments with pre-training a network based on RV and fine-tuning these weights to predict HR (compared to training with random weights) resulted in only a small performance increase (see Supplementary Section: Handling Missing Data During Training and Supplementary Fig. S1), and further experiments may be warranted. Commonly, transfer learning is performed by freezing the early layers (high-level features) and training the remaining layers (low-level features) to specialize on another task. Since our network architecture comprises only two levels, a bi-LSTM layer and linear layers, fine-tuning only the LSTM layer may not be enough to transfer the knowledge between RV and HR and needs further investigation with different architectures. This direction is of particular interest given that the measured HR signal is often noisy.

A valid concern, however, is whether the proposed joint learning method was able to distinguish between HR and RV variations. We observed a closer similarity between the predicted signals compared to the measured signals. There could be several explanations for this phenomenon. On the one hand, the model might be operating under the presumption that the distinctions between the two signals are nuanced, and/or the properties of the model architecture may be constraining the two reconstructed signals to be more similar than they truly are. On the other hand, noise distributions specific to the recording devices might diminish the correlation between the actual measured RV and HR signals. This noise component might be effectively filtered out by our joint learning model, hence the closer predicted correlation. Further research is warranted to understand the cause of this phenomenon.

Here, we adopted a large dataset to train and evaluate our models. Although an initial quality-check was performed on physiological recordings, post-visual inspection indicated that many still contained artifacts such as clipping and imperfect heart-beat detection. However, substantial improvement in performance beyond recent published work (from medians of approximately r ∼0.5 (Bayrak et al., 2020; Salas et al., 2021) to r ∼0.7 for RV in the current study) was attained. (Of note, fMRI data has low signal-to-noise ratio. Given that meaningful fluctuations comprise about 1–4% in the data, correlations at and above 0.5–0.6 between fMRI and an external measure are considered to be relatively strong in the neuroscience field.) In the previous studies, we used a small, visually inspected subset of the HCP-YA data consisting only of RV signals. The aforementioned improvement suggests that a larger, albeit less strictly vetted, dataset was effective for learning physiological patterns. Nonetheless, while a wide variety of signals (including some low-quality examples) may be advantageous during training, low-quality physiological data colors measurements of performance during testing, as these physiological recordings are taken as ground truth. In other words, low correlation scores can emerge when there are artifacts in the physiological recordings, necessitating close inspection as in Figure 8.

While much remains to be explored in terms of regional predictiveness of physiological signals beyond the current heuristic approach, the strong performance of tractography-based ROIs suggests that incorporating parcellation boundaries derived from other imaging modalities (e.g., diffusion-weighted MRI) and non-gray-matter regions could benefit fMRI prediction tasks. Through our analysis of individual ROI models, we found that the white matter regions within the brainstem (see the white matter atlas in Fig. 4a) were highly predictive of both breathing and heart rate. However, when comparing models with and without the inclusion of brainstem regions, no significant difference in overall performance was observed. The latter finding may suggest that the model was able to extract sufficient information from other distributed brain regions and compensate for the lack of these individually, highly predictive ROIs. Further, results from the percent ROI models (Section 3.4) demonstrate that performance could be boosted by increasing the number of ROIs that are included in the training. While this finding supports the need for comprehensive atlases with finer subdivisions, the exponentially decaying projection in Figure 5b suggests that dimensionality reduction using atlases sufficiently captures low-frequency physiological information. A model trained using the whole-brain average (global) signal as input was also found to yield promising performance (Supplementary Table S6).

As discussed in Section 3.6, models designed to decode physiological variations from fMRI may have the potential to ‘repair’ transient artifacts in the measured physiological data. This may be one factor that accounts for the generally larger amount of fMRI temporal variance explained by the predicted (compared to measured) physiological signals. Yet, it is also possible that our models, while designed to predict HR and RV from fMRI data, may also inadvertently decode neural features and/or other noise unrelated to HR and RV, increasing the variance explained compared to those of the measured physiological data. Moreover, the larger portion of variance explained might also arise from the fact that the decoded signals are themselves derived from the input fMRI data, albeit through a complex, nonlinear transformation.

The present work is directly inspired by reports showing that low-frequency physiological processes can impact fMRI analyses (Birn et al., 2006; Power et al., 2017; Wise et al., 2004; Xifra-Porxas et al., 2021). Consistent with prior work, we found that modeling physiological effects could alter maps of functional connectivity, and that RV and HR signals can account for substantial temporal variation in certain brain regions. Previous studies have also found that in task conditions, changes in cerebral oxygenation, captured in part by respiration and heart rate signals, may modulate the magnitude of observed task activation responses (Bright et al., 2020). These findings suggest that accounting for RV and HR signals can advance individual-level precision in neuroscience and medicine using fMRI. As such, it is important to have general, versatile models that can reconstruct these features in the common scenario of missing or noisy physiological recordings. Notably, in addition to its use for ‘denoising’ - i.e., removing RV and HR effects from fMRI signals, the proposed method may also open possibilities for studying *neural* processes relating to autonomic regulation in data where physiological signals have not been monitored. Specifically, we may expect that a component of the reconstructed RV and HR signals may track large-scale neural activity that is linked with cortical autonomic control (e.g., Tu & Zhang, 2022).

The current results suggest a number of future directions. One springs from limitations in the use of population-level atlases for defining fMRI parcellations. The present study used atlases that had been derived from populations, yet individuals can exhibit variability in the spatial boundaries of functional regions (Kong et al., 2021). Thus, individual- or cohort-specific parcellations may enable higher precision in extracting personalized physiological information from fMRI. The issue of precision is amplified when working with datasets from different stages of human life, including infant or aging datasets, which do not conform with the topographical features of the young cohort that is used to train networks in this work.

Additionally, while the current work focused on developing resting-state and task models using HCP-YA datasets, future research should focus on validating and enhancing the generalizability of the models across diverse samples, such as by including various age groups, different physiological events (capturing variable breathing patterns such as deep breathing and breath-holding, and unintentional apneas), and different fMRI paradigms. Incorporating diverse features into training datasets could substantially enhance model generalizability and potentially improve the predictive accuracy.

An important consideration in studies examining the relationship between physiological signals and BOLD fluctuations is the sampling rate of the fMRI data. Variations in heart rate can occur at low frequencies (e.g., < 0.15 Hz), and have been shown to strongly correlate with BOLD signal fluctuations within this range (Akselrod et al., 1981; Cohen & Taylor, 2002; Otzenberger et al., 1998; Shmueli et al., 2007). Although shorter repetition times allow for capturing higher-frequency physiological fluctuations, the TRs used in this study (ranging from 0.72 - 2.1 s) are likely sufficient for capturing the low-frequency heart rate fluctuations that are the focus of this analysis. However, these temporal resolutions still impose limitations on the precision with which heart rate variability can be assessed. As a result, the ability to isolate and characterize cardiac-related components in the BOLD signal may be constrained.

Another limitation that can be addressed in future work is model interpretability, a common problem with machine learning models. Methods such as Shapley Values (Ghorbani & Zou, 2019) can offer explanations for model predictions and provide insights into the model development process. Architectural modularity (multiple layers) could be considered to allow transfer learning (Chollet, 2021), since the ability to freeze layers that capture high-level information may further boost the performance of joint learning.

## 5 Conclusions

Modeling physiological variability is increasingly recognized as crucial for both (1) improving the sensitivity of fMRI to neural effects, as well as (2) providing valuable information about cerebrovascular health, brain states, and emotion regulation. Since high-quality physiological signals are often missing from fMRI datasets, the present study fills this gap by introducing a generalizable tool for reconstructing two key physiological signals (RV, HR) from fMRI data. The proposed framework was found to be robust across a broad range of experimental conditions and imaging protocols, indicating that it can enrich a broad array of fMRI datasets with missing physiological information. This study motivates future work on methodological advances for modeling RV/HR, and enables retrospective studies of physiological effects in health and disease, leveraging the large body of fMRI databases that have been acquired without physiological signals.

## Supplementary Material

Supplementary Material

## Data Availability

Dataset 1 and Dataset 2 used in the present study are available for download from the Human Connectome Project (www.humanconnectome.org). Dataset 3 is available for download from OSF (https://osf.io/wzakd/). Moreover, pretrained model weights and predicted RV and HR signals are publicly available; further information is provided in the documentation at https://github.com/neurdylab/deep-physio-recon. *DeepPhysioRecon* model checkpoints can be found on huggingface (https://huggingface.co/rgbayrak/deep-physio-recon). Inference and all relevant source code can be found on GitHub (https://github.com/neurdylab/deep-physio-recon), along with user documentation. The code for data pre-/post-processing and analysis is shared and illustrates the use of *DeepPhysioRecon* using example data.
