# Supplementary material for: DeepPhysioRecon: Tracing peripheral physiology in low frequency fMRI dynamics
Source: Imaging Neurosci (Camb). 2025 Sep 25;3:IMAG.a.163. doi: 10.1162/IMAG.a.163 (PMC12464743; doi:10.1162/IMAG.a.163)
Supplement: Supplementary Material [file IMAG.a.163_supp.pdf]

1 Supplementary information

1.1 Handling Missing Data During Training

The goal of training models that jointly learn RV and HR was to exploit the close coupling between RV and HR. Yet often times, only one of these physiological signals is acquired, and/or one of the signals is corrupted at particular times during the recording. Thus, we also ask whether RV and HR share common features that could enable the models to be trained on datasets that contain only one of these signals, and yet still be able to predict both (RV and HR) signals. Two experiments are designed to answer this question. In the first, we control the respective contributions of RV and HR to the loss function, and in the second, we simulate missing data.

To control the contribution of RV and HR to model training, we pursue a model training strategy whereby the weighting of each term in the loss function is varied while the model is trained to jointly estimate both signals. To this end, we include a hyperparameter λ which governs the respective contributions of each term. As shown in Supplementary Figure 1a, even a small contribution from either term (when λ is 0.0001) is sufficient to learn the corresponding waveform. When there is no contribution from one of the terms (i.e. $\lambda = 0$, corresponding to no RV, or $\lambda = 1$, corresponding to no HR), the models did poorly in learning the signal (RV or HR) whose input was not taken into account (see Supplementary Figure 1a).

$$L = \lambda * L_{RV} + (1 - \lambda) * L_{HR} \tag{1}$$

In the next experiment, we asked whether we could predict HR, which tends to be noisier than RV, based on a model trained primarily with RV signal outputs. To simulate this scenario, we pretrained a model using all available RV data and fine-tuned these models using a varying number of HR labels, which imitated the case of missing HR data. We find (Supplementary Figure 1b) that the models indeed benefited from initialization with pretrained RV-weights in the case where limited HR labels were made available in the training. As expected, the advantage was less pronounced when more HR labels were used.

1.2 Percent variance explained (pve) by measured and predicted waveforms

1.2.1 Regional distribution

To demonstrate the regional distribution of variance explained by RV and HR, we average percentage variance explained maps within each Findlab subnetworks across subjects. For each ROI, we create pairs of box plots: one for measured and one for predicted. Each box plot represents the PVE averaged within an ROI for each subject, and the aggregate values are visualized to highlight the differences and distributions across ROIs, as shown in Supplementary Figure 2.

1.2.2 Intra-subject inter-scan repeatability

To examine the relationship between measured and predicted RV/HR time-series, we conducted an analysis comparing the variance explained maps from each scan within subjects (4 scans per subject in Dataset 1) for both measured and predicted data. Specifically, for each subject, we computed the similarity between each possible pair of their 4 variance explained maps (six unique comparisons per subject), excluding self-comparisons, and then averaged these similarity values. The results show that the variance explained maps calculated using the predicted RV/HR waveforms exhibited higher inter-scan repeatability, see Supplementary Figure 3. Indeed, the findings support our earlier hypothesis that the models may clean the physiological waveforms and potentially improve test-retest reliability.

1.3 Impact of predicted signals on functional connectivity at the group level

1.3.1 Functional Connectivity

Here, we determine brain regions whose functional connectivity, at the group level, is significantly altered by modeling physiological information, comparing the use of measured versus predicted physiological signals. First, we conduct t-tests between functional connectivity values at each pair of ROIs, calculated before versus after removing **measured** physiological signals from fMRI data, and then repeat the experiment for **predicted** physiological signals. We provide the resulting p-values to a multiple comparison test, the false discovery rate (FDR) (Genovese et al., 2002). The resulting maps are binarized using threshold 0.05 and shown in Supplementary Figure 4. We observed that, despite a high degree of individual variability in the influence of physiological regression on FC, the group-level FC matrices were similar in many regions except those indicated in this plot.

1.3.2 Seed-based Connectivity

We conducted three paired t-test comparisons: between seed-based FC maps calculated (1) after measured versus predicted physiological regression; (2) before versus after measured physiological regression; and (3) before versus after predicted physiological regression. For each comparison, we control for multiple comparisons using FDR correction (Genovese et al., 2002) with p ¡ 0.001. The resulting statistical maps (Supplementary Figure 5) show voxels where seed-based FC significantly differs between conditions. In each map, positive t-statistics (red/yellow) indicate regions where FC with the seed is higher in the second condition compared to the first, while negative t-statistics (blue) indicate regions where FC is reduced. As expected, comparisons (2) and (3) showed similar patterns: removing physiological signals (both measured and predicted) generally reduced positive correlations throughout the brain and appeared to weaken negative correlations in the ventricles. Comparison (1) showed positive effects, indicating removing our predicted physiological signals tends to reduce the connectivity more than removing the measured signals, which is consistent with the larger percent variance explained by the predicted signals (see Section 3.6).

1.4 Impact of head motion on HR prediction

As discussed in the main manuscript, when head motion is removed, the pseudo head motion caused by respiration is also removed, potentially losing important information for reconstructing respiratory signals. However, retaining it may obscure fMRI signals related to heart rate. While a more detailed exploration is warranted to conclusively characterize its impact on modeling physiological signals, we conducted a mini experiment. Briefly, we assessed the potential relationship between a subject's overall motion and the performance of HR prediction. Here, we utilized the motion statistics for these scans, specifically the average RMS values. The results Supplementary Figure 9 indicated a subject's movement was not a significant factor influencing the reliability of HR predictions.

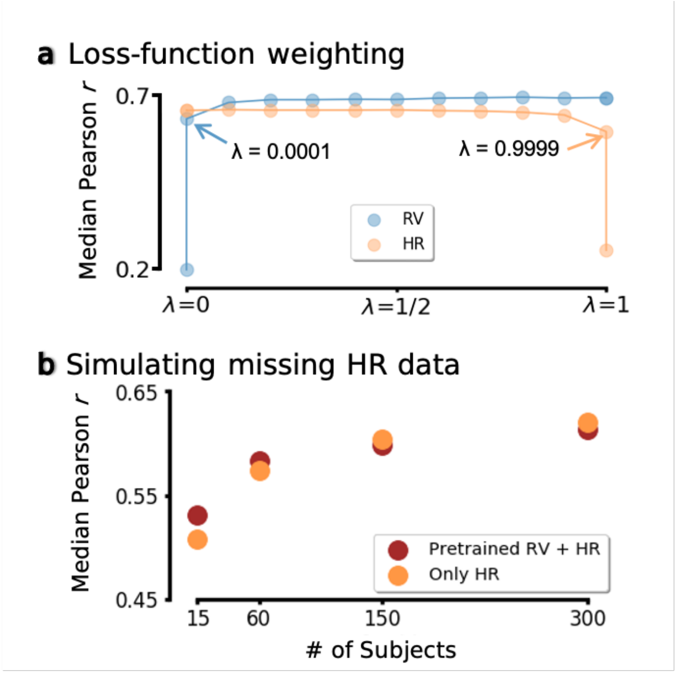

Supplementary Figure 1: (a) **Dependence of prediction accuracy on loss-function weighting.** For the deep bi-LSTM model, 13 models with different weights λ governing loss terms were trained (note the unequal divisions of the x-axis). Prediction performance is calculated as the mean Pearson correlation coefficient across the test set. (b) **Simulating missing HR data.** Models are trained with the goal of estimating HR signals in the scenario of missing or few HR recordings during fMRI scans. Model performance is shown when using a varying number of HR labels together with pretrained RV weights (red); or when using a varying number of HR labels without pretrained RV weights (orange).

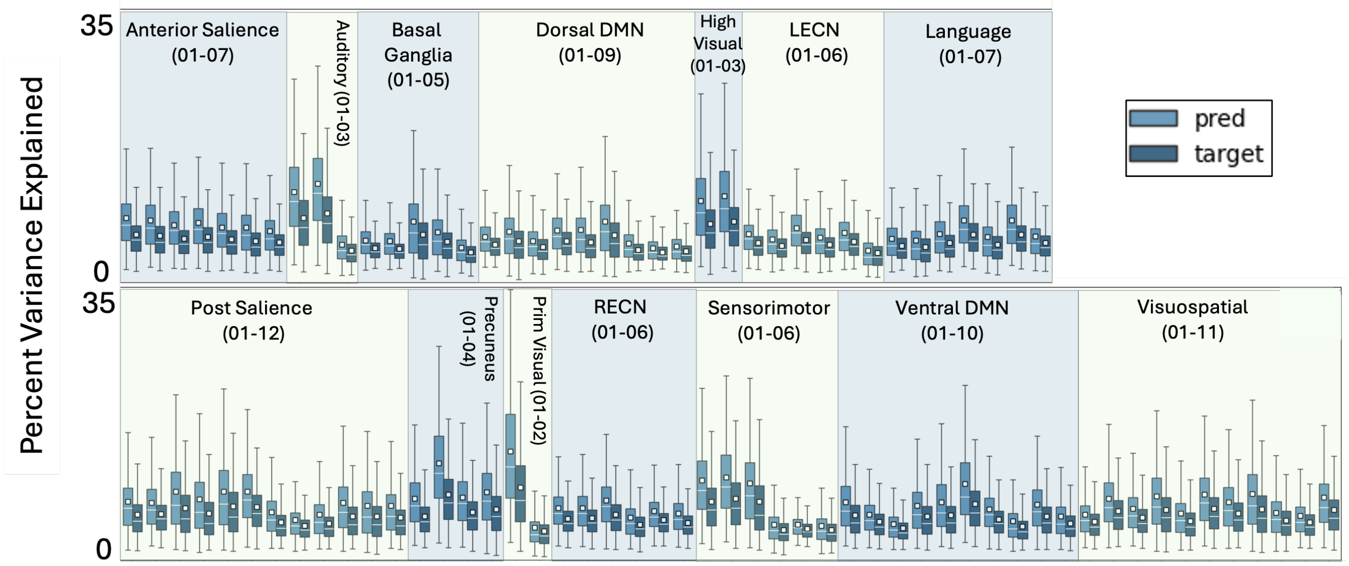

Supplementary Figure 2: Each box plot represents the PVE averaged within an ROI for each subject, highlighting differences and distributions across the ROIs.

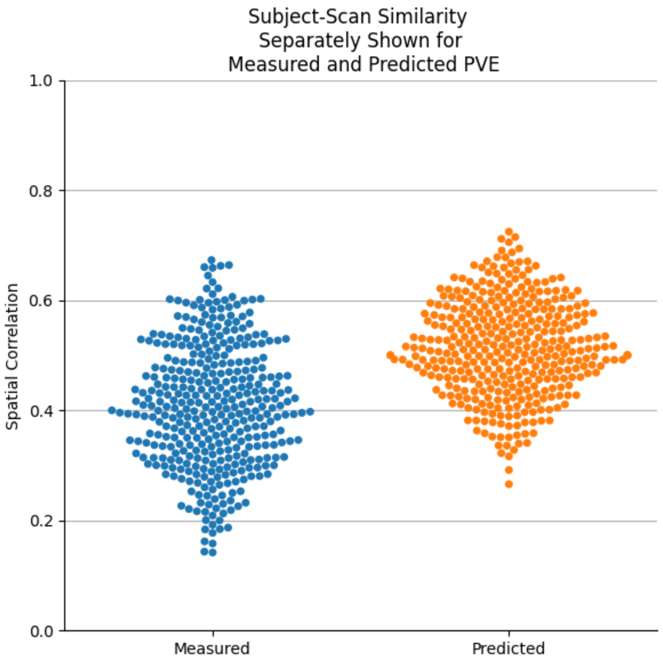

Supplementary Figure 3: Comparing the variance explained maps from each scan within subjects for both measured and predicted data.

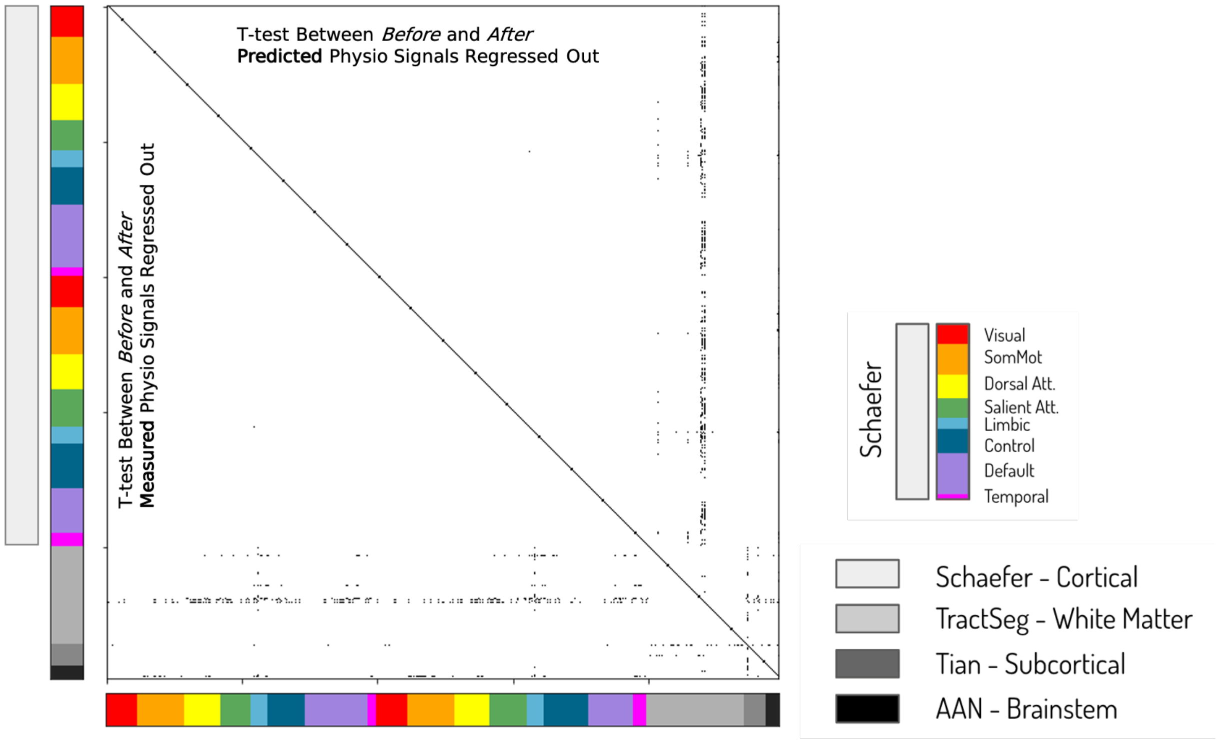

Supplementary Figure 4: Effects of regressing out the measured (lower triangle) and predicted (upper triangle) physiological signals on group-level functional connectivity. First, we conduct t-tests on functional connectivity values at each pair of ROIs, calculated before versus after removing **measured** physiological signals from fMRI data, and then repeat the experiment for **predicted** physiological signals. The resulting values are binarized at the statistical significance threshold of $p<0.05$, with false discovery rate (FDR) correction for multiple comparisons.

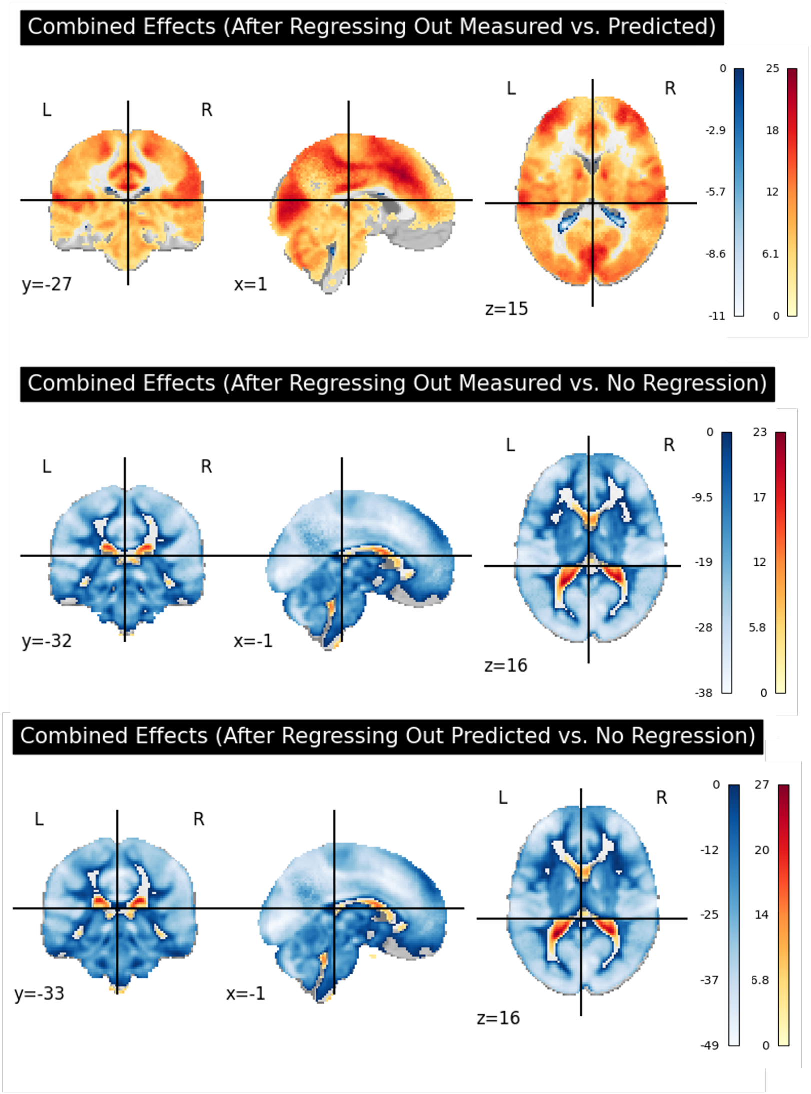

Supplementary Figure 5: Effects of regression on seed-based functional connectivity. Statistical maps (FDR-corrected $p < 0.001$) show areas where FC differs between conditions: (Condition 1, first row) measured versus predicted regression, where positive t-statistics indicate larger reduction in FC by predicted signals; (Conditions 2,3, bottom 2 rows) before versus after physiological regression (measured and predicted), where removing physiological signals reduced positive correlations and weakened negative correlations in ventricles.

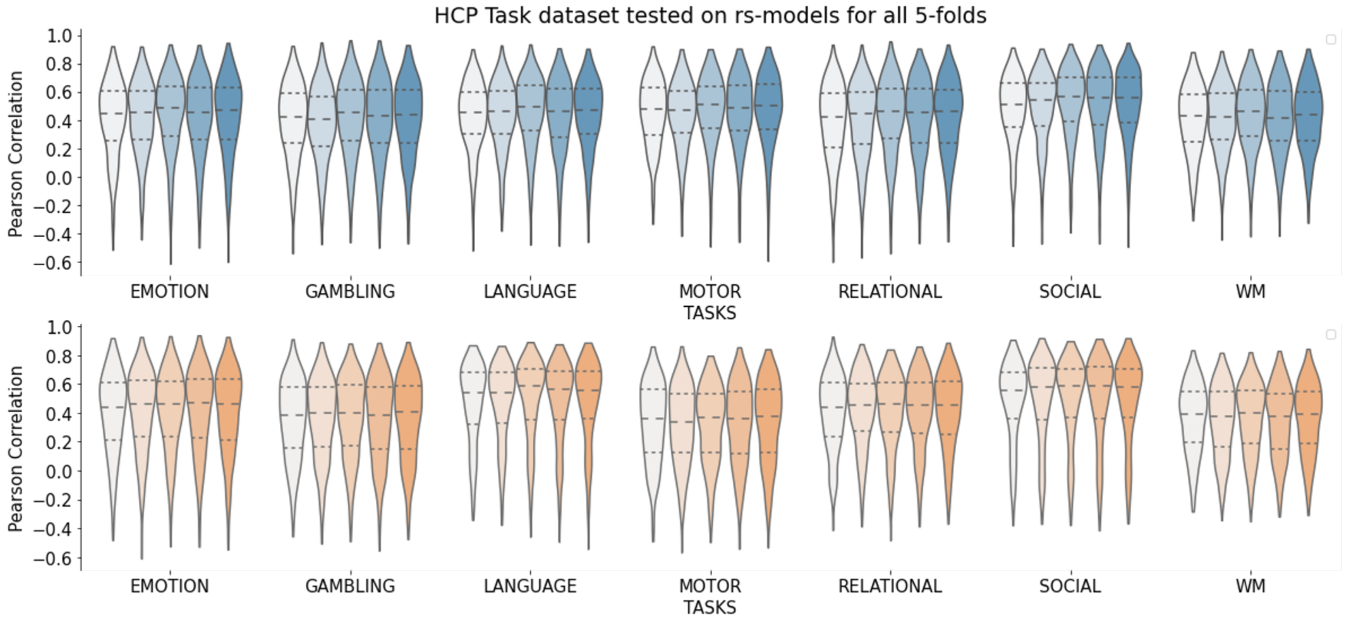

Supplementary Figure 6: Prediction of physiological signals in the HCP task dataset, using each of the 5 models produced by 5-fold CV on the training (HCP resting-state) data. Similar performance across the 5 folds indicates that the training resulted in reliable models. RV – shown in blue, HR – shown in orange.

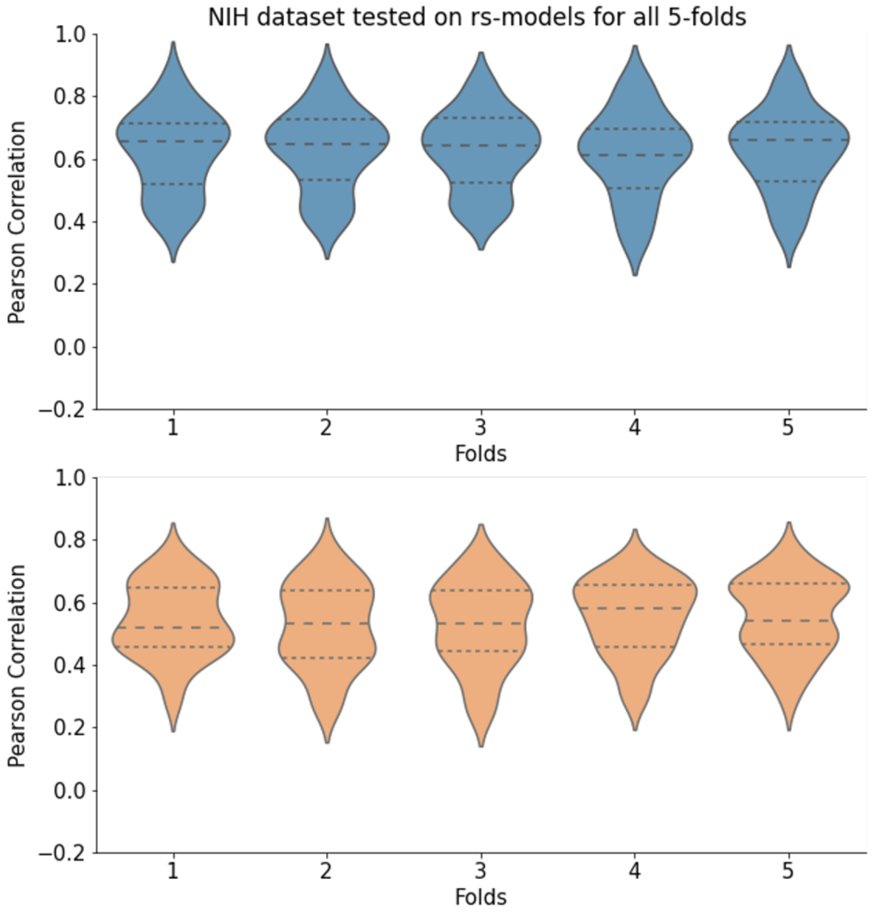

Supplementary Figure 7: Prediction of physiological signals in the in-house dataset, using each of the 5 models produced by 5-fold CV on the training (HCP resting-state) data. RV – shown in blue, HR – shown in orange.

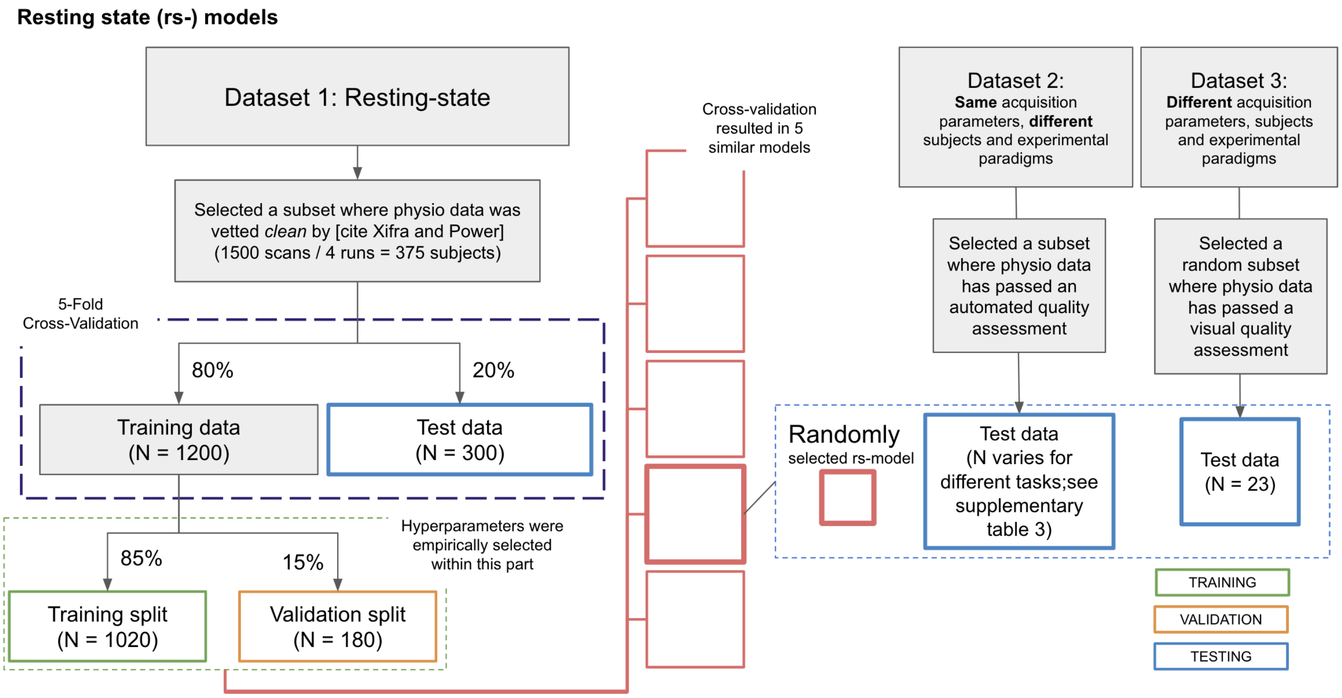

Supplementary Figure 8: Resting state model training.

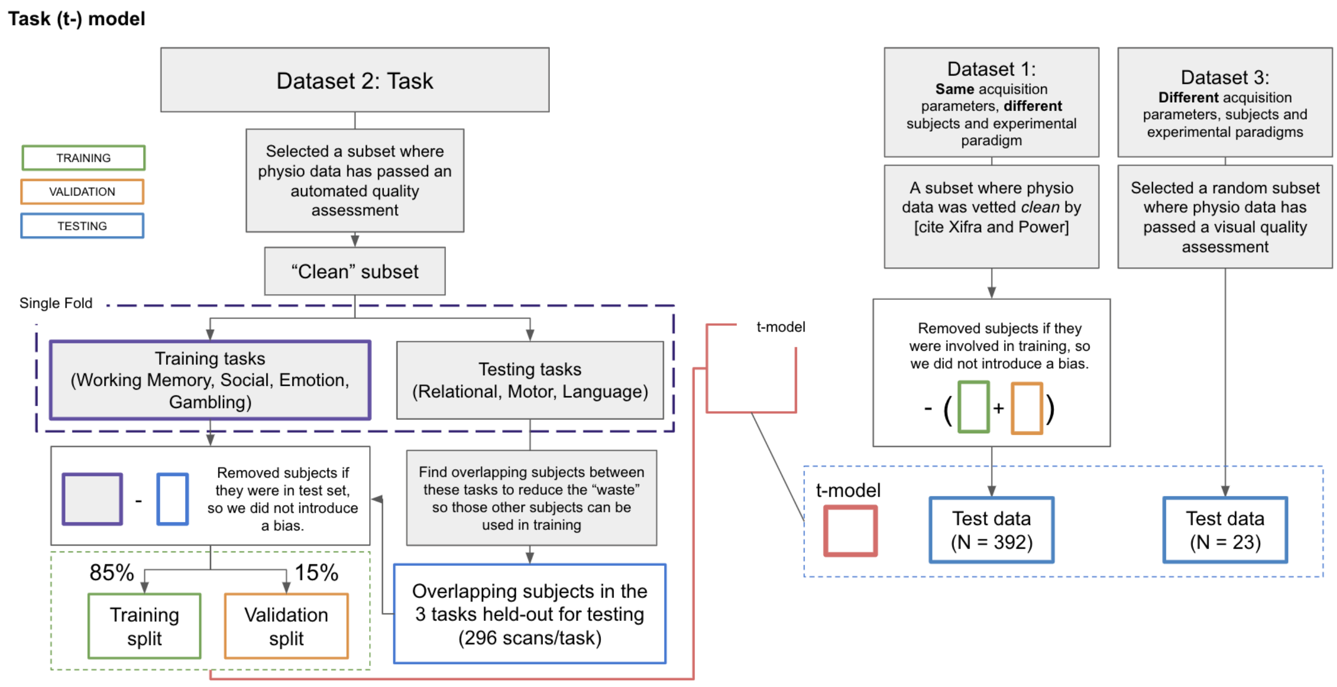

Supplementary Figure 9: Task model training.

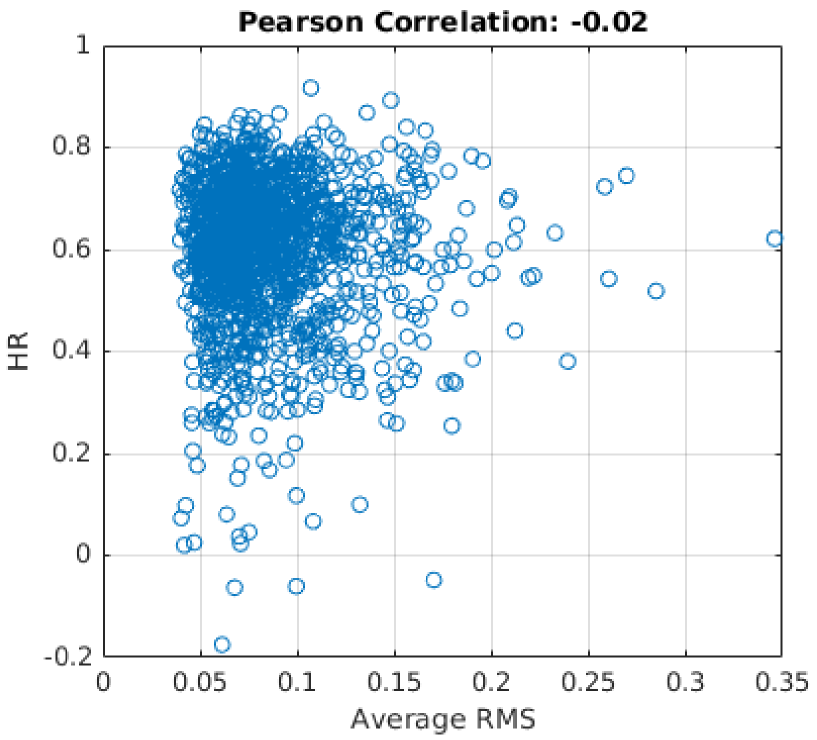

Supplementary Figure 10: Correlation between gross motion (RMS) and HR prediction accuracy.

References

Genovese, C. R., Lazar, N. A., & Nichols, T. (2002). Thresholding of statistical maps in functional neuroimaging using the false discovery rate. *Neuroimage*, *15*(4), 870–878.

	Testing			
	Training	Held-out	Out-of-sample	Out-of-distribution
Resting State Model	Resting State	Resting State	Working Memory, Social Cognition, Emotion Processing, Gambling, Relational Processing, Motor, Language	In-house
Task Model	Working Memory, Social Cognition, Emotion Processing, Gambling	Relational Processing, Motor, Language	Resting State	In-house

Supplementary Table 1: Datasets used for training and testing our models.

Resting State	Working Memory	Social Cognition	Emotion Processing	Gambling	Relational Processing	Motor	Language	In-house
1500	977	1018	1209	1136	1027	649	976	23

Supplementary Table 2: Number of scans that passed quality checks.

Training	Testing							
Resting State	Working Memory	Social Cognition	Emotion Processing	Gambling	Relational Processing	Motor	Language	In-house
1200	620	634	735	716	645	394	632	23

Supplementary Table 3: Training and testing data for resting state (rs-) models.

Training				Testing				
Working Memory	Social Cognition	Emotion Processing	Gambling	Relational Processing	Motor	Language	Resting State	In-house
652	664	840	797	296	296	296	392	23

Supplementary Table 4: Training and testing data for task (t-) model.

	RV			HR		
	Relational Processing	Motor	Language	Relational Processing	Motor	Language
rs-model	0.448	0.469	0.488	0.453	0.559	0.362
t-model	0.552	0.474	0.534	0.516	0.600	0.458

Supplementary Table 5: Model performance comparison between resting state (rs-) and task (t-) models when applied to task data. Performance is measured as the Pearson correlation score between measured and predicted physiological signals in test set. Median scores are reported in this table.

	Pearson R (Median, IQR)	
Input	RV	HR
Baseline ROIs	0.6909 (0.2170)	0.6157 (0.1637)
Global Signal	0.5442 (0.2425)	0.4800 (0.1914)

Supplementary Table 6: Model performance (Pearson's R; median and interquartile range, IQR) for respiration volume (RV) and heart rate (HR) prediction across 5-fold cross-validation. Results are reported for the resting-state model (Base) and the global signal model (GS). *The software has since been updated and the resting-state model was rerun using the same train/test fold splits. Slight numerical differences from earlier reported values (previously median R = 0.679 for RV, 0.625 for HR) are expected, as stochastic optimization may converge to slightly different local optima across runs, even with identical splits.
